# *Pseudomonas aeruginosa* mutants defective in glucose uptake have pleiotropic phenotype and altered virulence in non-mammal infection models

**DOI:** 10.1038/s41598-018-35087-y

**Published:** 2018-11-15

**Authors:** Matteo Raneri, Eva Pinatel, Clelia Peano, Giordano Rampioni, Livia Leoni, Irene Bianconi, Olivier Jousson, Chiara Dalmasio, Palma Ferrante, Federica Briani

**Affiliations:** 1 Dipartimento di Bioscienze, Università degli Studi di Milano, Milano, Italy; 2 Istituto di Tecnologie Biomediche-CNR, Segrate, Italy; 3 Dipartimento di Scienze, Università degli Studi Roma Tre, Roma, Italy; 4 Centre for Integrative Biology, Università degli Studi di Trento, Trento, Italy; 5 Present Address: Istituto Clinico Humanitas-CNR, Rozzano, Italy

## Abstract

*Pseudomonas* spp. are endowed with a complex pathway for glucose uptake that relies on multiple transporters. In this work we report the construction and characterization of *Pseudomonas aeruginosa* single and multiple mutants with unmarked deletions of genes encoding outer membrane (OM) and inner membrane (IM) proteins involved in glucose uptake. We found that a triple Δ*gltKGF* Δ*gntP* Δ*kguT* mutant lacking all known IM transporters (named GUN for Glucose Uptake Null) is unable to grow on glucose as unique carbon source. More than 500 genes controlling both metabolic functions and virulence traits show differential expression in GUN relative to the parental strain. Consistent with transcriptomic data, the GUN mutant displays a pleiotropic phenotype. Notably, the genome-wide transcriptional profile and most phenotypic traits differ between the GUN mutant and the wild type strain irrespective of the presence of glucose, suggesting that the investigated genes may have additional roles besides glucose transport. Finally, mutants carrying single or multiple deletions in the glucose uptake genes showed attenuated virulence relative to the wild type strain in *Galleria mellonella*, but not in *Caenorhabditis elegans* infection model, supporting the notion that metabolic functions may deeply impact *P*. *aeruginosa* adaptation to specific environments found inside the host.

## Introduction

*Pseudomonas aeruginosa* is a Gram-negative ubiquitous bacterium with high metabolic versatility, able to thrive in disparate environments and to infect hosts as different as plants, insects and mammals. However, the number of sugars that *P*. *aeruginosa* (and other species belonging to the *Pseudomonas* genus) can exploit as carbon and energy sources is relatively low and substantially limited to glucose, glucuronic acid and fructose^1^.

**Figure 1:**
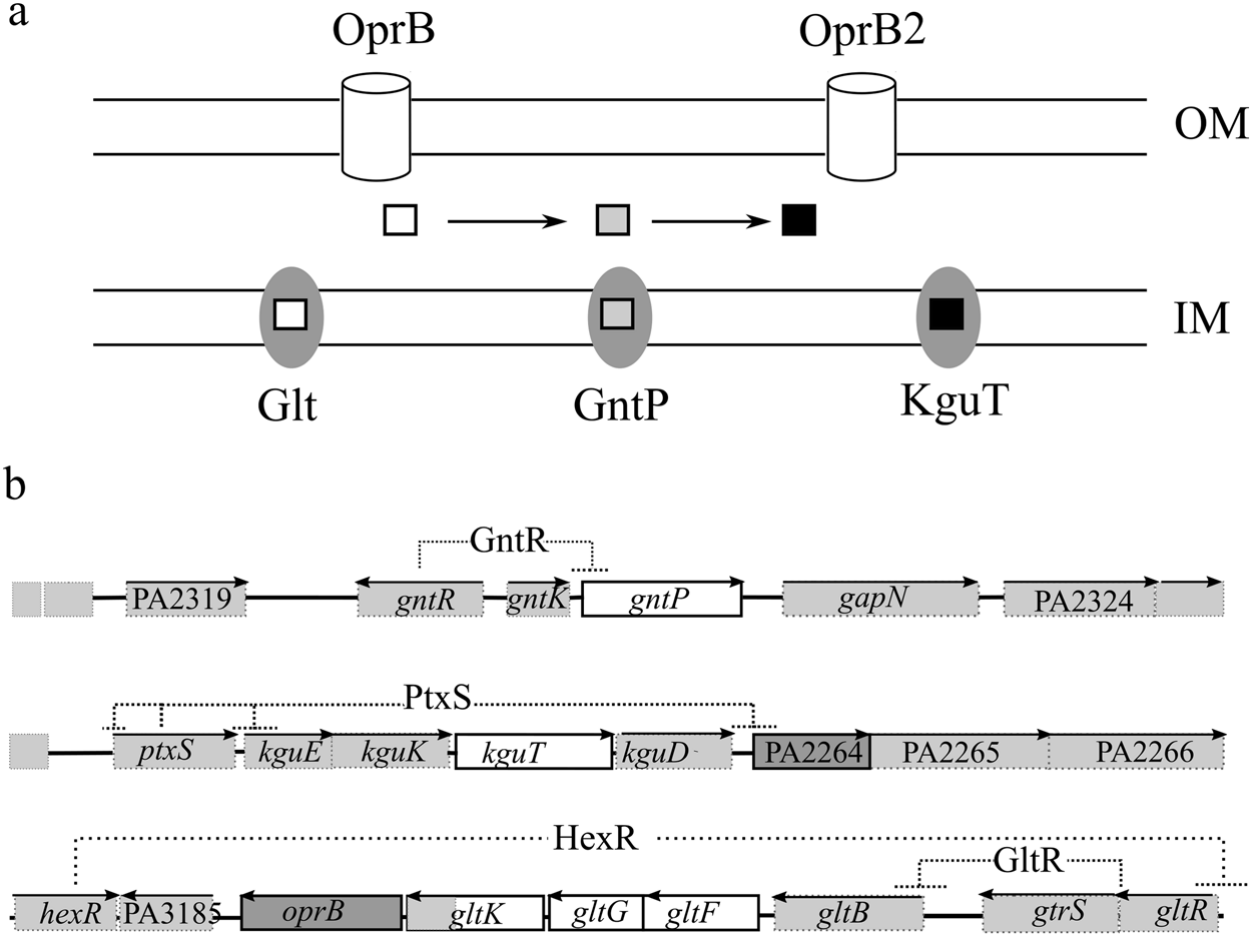
Glucose uptake pathways and organization of glucose uptake genes on *P*. *aeruginosa* genome. (**a**) Different porins allow glucose (empty square) entry into the periplasm. Once in the periplasm, glucose can be transported into the cytoplasm by Glt or oxidized to gluconate (grey square) and 2-KG (black square). Gluconate and 2-KG enter the cytoplasm thanks to GntP and KguT. Cylinders, outer membrane (OM) porins implicated in glucose uptake; ovals, inner membrane (IM) transporters. (**b**) Organization and regulation of glucose uptake genes in *P*. *aeruginosa* PAO1. 10 kbp long genomic regions encompassing the deleted loci encoding IM and OM proteins (represented as empty and dark boxes, respectively) are schematized. Boxes, ORFs; lines, intergenic regions; arrows on top of boxes indicate the transcription direction. Dashed lines connect genes encoding negative regulators with their target promoter(s).

*Pseudomonas* glucose uptake pathway was first clarified in *P*. *putida*^2,3^. Homologues of *P*. *putida* transporters were identified in *P*. *aeruginosa* and in some cases, shown to be *bona fide* orthologues (i.e. endowed with the same function) of *P*. *putida* proteins. Once crossed the outer membrane through the OprB porin, glucose can either cross the inner membrane thanks to the ABC transporter Glt or enter a periplasmic oxidative pathway producing gluconate and 2-ketogluconate (2-KG)^4,5^. Gluconate and 2-KG are internalized into the cytoplasm by the specific transporters GntP and KguT, respectively (Fig. 1a). In the cytoplasm, the two import pathways converge on the synthesis of 6-phosphogluconate, which enters the Entner-Doudoroff pathway^3^. The two alternate pathways (i.e. the Glt- dependent phosphorylative pathway and the GntP and KguT-dependent oxidative one) are regulated by glucose availability, with the oxidative route being preferentially used under high glucose^1,6,7^. Different regulators modulate the expression of the operons encoding Glt, GntP and KguT (Fig. 1b). The *gntP* gene and the *kgu* operon are negatively regulated by the GntR and PtxS repressors, respectively, whereas *glt-oprB* transcription is negatively modulated by the GltR response regulator, whose activity is controlled by the histidine kinase GtrS^8^. In the presence of glucose, repression by all these regulators is relieved (Fig. 1b)^1,5,8–11^. Interestingly, GtrS and PtxS regulate also transcription of *toxA*, the gene encoding the key virulence factor exotoxin A, thus establishing a link between glucose metabolism and virulence^8–10,12^.

Metabolic genes have been repeatedly identified in *in vivo* screenings for *P*. *aeruginosa* functions contributing to virulence^13,14^, suggesting that the inhibition of metabolic pathways may represent a sound strategy for the development of antibacterial therapies. In this respect, factors involved in glucose transport and utilization deserve consideration. In fact, pathologies leading to increased glucose concentration in the host fluids, from blood to liquid covering the respiratory mucosae, determine an augmented risk of developing serious bacterial infections not only for the immunity dysfunction due to hyperglycemia, but also because of glucose-dependent stimulation of bacterial growth^15–18^. Interfering with the ability of pathogenic bacteria to import or use glucose could thus represent a promising antibacterial strategy^19^.

In this work, we report the generation of a collection of *P*. *aeruginosa* mutants with unmarked deletions in genes encoding the outer membrane (OM) OprB and PA2291 porins or the inner membrane (IM) Glt, GntP and KguT transporters. Deletions of the genes for porins and for the IM oxidative route transporters were combined in the double Δ*oprB* ΔPA2291 and Δ*gntP* Δ*kguT* mutants, respectively. A triple Δ*glt* Δ*gntP* Δ*kguT* mutant lacking all IM transporters was also generated and characterized. The mutants were assayed for phenotypes linked to *P*. *aeruginosa* virulence^20^. Interestingly, mutants lacking the oxidative route of glucose import showed altered virulence in the *Caenorhabditis elegans* and *Galleria mellonella* infection models.

## Results

### Generation of *P. aeruginosa* mutants defective in glucose uptake

We constructed single mutants of *P*. *aeruginosa* PAO1 lacking the genes for the OM porin OprB, its paralogue PA2291 (encoding OprB2, a porin 95% identical to OprB)^21^, the IM putative gluconate permease GntP or the 2-KG transporter KguT. Single mutations were combined in order to generate strains defective for both *oprB* and PA2291, or both *gntP* and *kguT* genes of the oxidative route of glucose transport. We also inserted a Δ*gltKGF* mutation, eliminating *gltG*, *gltF* and part of *gltK*, both in PAO1 and in the double mutant Δ*gntP* Δ*kguT*, obtaining *P*. *aeruginosa* strains devoid of Glt only or lacking all the Glt, GntP and KguT IM transporters (Fig. 1b; Supplementary Table S1).

**Figure 2:**
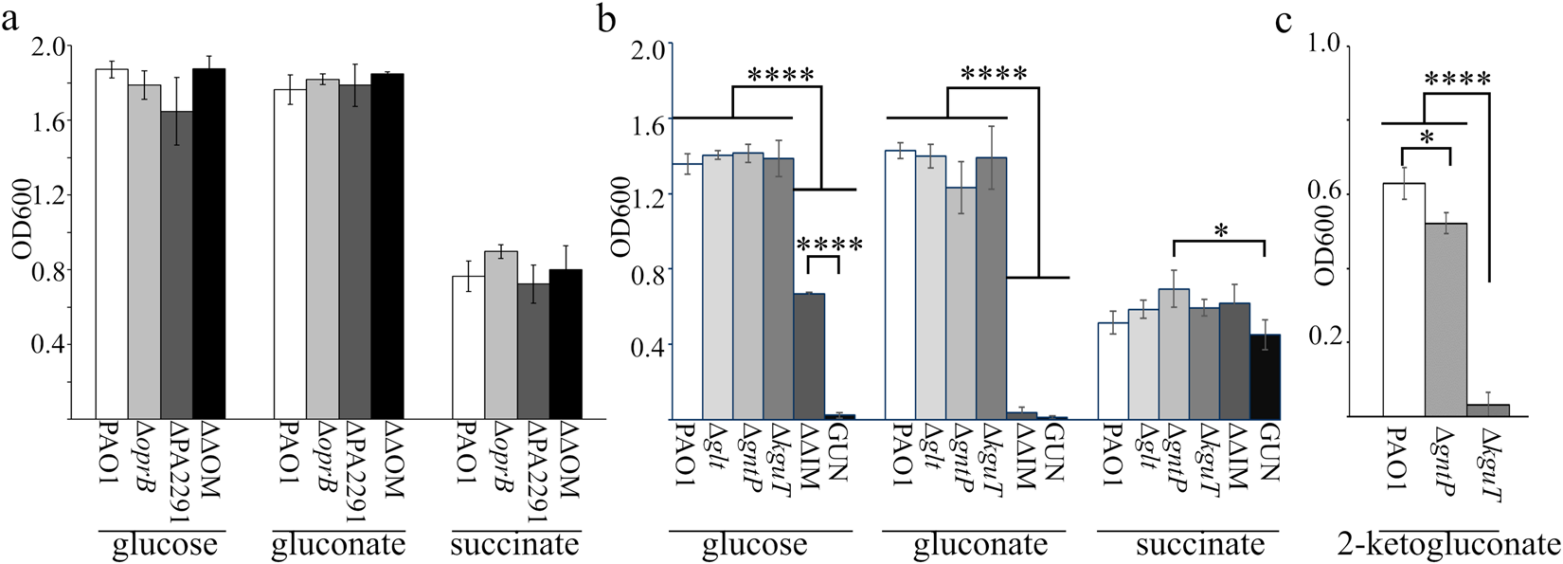
Growth of glucose uptake mutants with different carbon sources. PAO1 and mutant strains were inoculated at the same OD_600_ in M9-Triton X-100 supplemented with either 0.4% glucose, 0.4% gluconate, 0.1% 2-ketogluconate or 0.5% succinate, as indicated below the bars. Bacterial cell density (OD_600_) was measured after aerate incubation for 24 h at 37 °C. The bars represent average (n = 3) with standard deviation (SD). (**a**) OM mutants. ΔΔOM, Δ*oprB* ΔPA2291 mutant. (**b**). IM mutants. ΔΔIM, Δ*gntP* Δ*kguT* mutant. (**c**). Growth in 0.1% 2-ketogluconate of the indicated mutants. (**a**,**b**) Significance was evaluated with One-way Anova and Tukey post-hoc test. (**a**) According to one-way Anova analysis, growth differences were not statistically significant at 0.05 level. (**b**,**c**). *P < 0.5; ****P < 0.0001. All comparisons for which P is not shown were not significant at 0.05 level.

The growth curves and optical density reached by the mutants after 24 hours of incubation in minimal medium supplemented with glucose or gluconate were analysed. As a control, growth in succinate was monitored, since succinate uptake depends on other transporters^22^. Both single and double mutants lacking the OM OprB and/or OprB2 did not show any growth difference with respect to PAO1 (Supplementary Fig. S1) and in the optical density reached after 24 h of growth (Fig. 2a) in the tested media. These results indicate that the OprB and OprB2 porins are dispensable for glucose utilization, and that glucose can likely pass the OM through other porins. Therefore, we did not further characterize the mutants lacking OprB or/and OprB2, and we focused our analyses on mutants lacking IM transporters.

Single IM mutants and PAO1 reached comparable optical density after 24 h incubation in the tested media (Fig. 2b). However, the Δ*gntP* mutant had a longer lag phase in glucose and gluconate and lower growth rate in the latter medium (Table 1; Supplementary Fig. S1). To discriminate the roles of GntP and KguT in the transport of gluconate and 2-KG, the growth of the Δ*gntP* and Δ*kguT* mutants in minimal medium supplemented with 2-KG was analysed. The Δ*gntP* mutant was able to grow on 2-KG as sole carbon source, whereas Δ*kguT* was not (Fig. 2c), thus confirming that 2-KG enters the cytoplasm only through the KguT transporter^5^.

**Table 1:** Generation time of glucose uptake mutants.

Strain^b^	Generation time (min)^a^		
	Glucose^c^	Gluconate^c^	Succinate
PAO1	36.7 ± 2.8^(a)^	34.1 ± 3.6^(a)^	36.4 ± 3.9^(ab)^
Δ*glt*	39.5 ± 1.6^(a)^	36.1 ± 2.2^(a)^	39.9 ± 1.9^(ab)^
Δ*gntP*	40.5 ± 1.1^(a)^	53.4 ± 7.2^(b)^	32.6 ± 2.4^(^^b)^
Δ*kguT*	38.6 ± 5.7^(a)^	39.3 ± 3.1^(a)^	43.5 ± 3.7^(ab)^
Δ*gntP* Δ*kguT*	64.2 ± 11.6^(b)^	na	48.3 ± 4.6^(a)^
GUN	na	na	48.8 ± 8.6^(a)^

^a^Mean values (n = 3) with SD of generation time in exponential growth phase. Cultures were grown as described in Supplementary Fig. S1 legend. The differences between means reported in the same column and sharing at least one letter (in brackets) were not statistically significant according to One-way ANOVA and Tukey post-hoc test.

^b^PAO1 single and double mutants are indicated by their cognate mutation(s).

^c^na, not applicable. No OD_600_ increase detectable in the timespan of the experiments (7 h).

As expected, the multiple mutants exhibited stronger growth defect in glucose/gluconate media. In fact, the Δ*gntP* Δ*kguT* double mutant showed reduced growth in glucose and did not grow in gluconate. Finally, the Δ*glt* Δ*gntP* Δ*kguT* strain was unable to grow in either glucose or gluconate, showing that no transporters other than Glt, GntP and KguT operate glucose uptake in *P*. *aeruginosa* (Table 1; Fig. 2b). Because of its inability to grow with glucose as sole carbon source, the Δ*glt* Δ*gntP* Δ*kguT* mutant was named GUN (i.e. Glucose Uptake Null).

### Lack of glucose uptake systems deeply affects *P*. *aeruginosa* transcription profile

**Figure 3:**
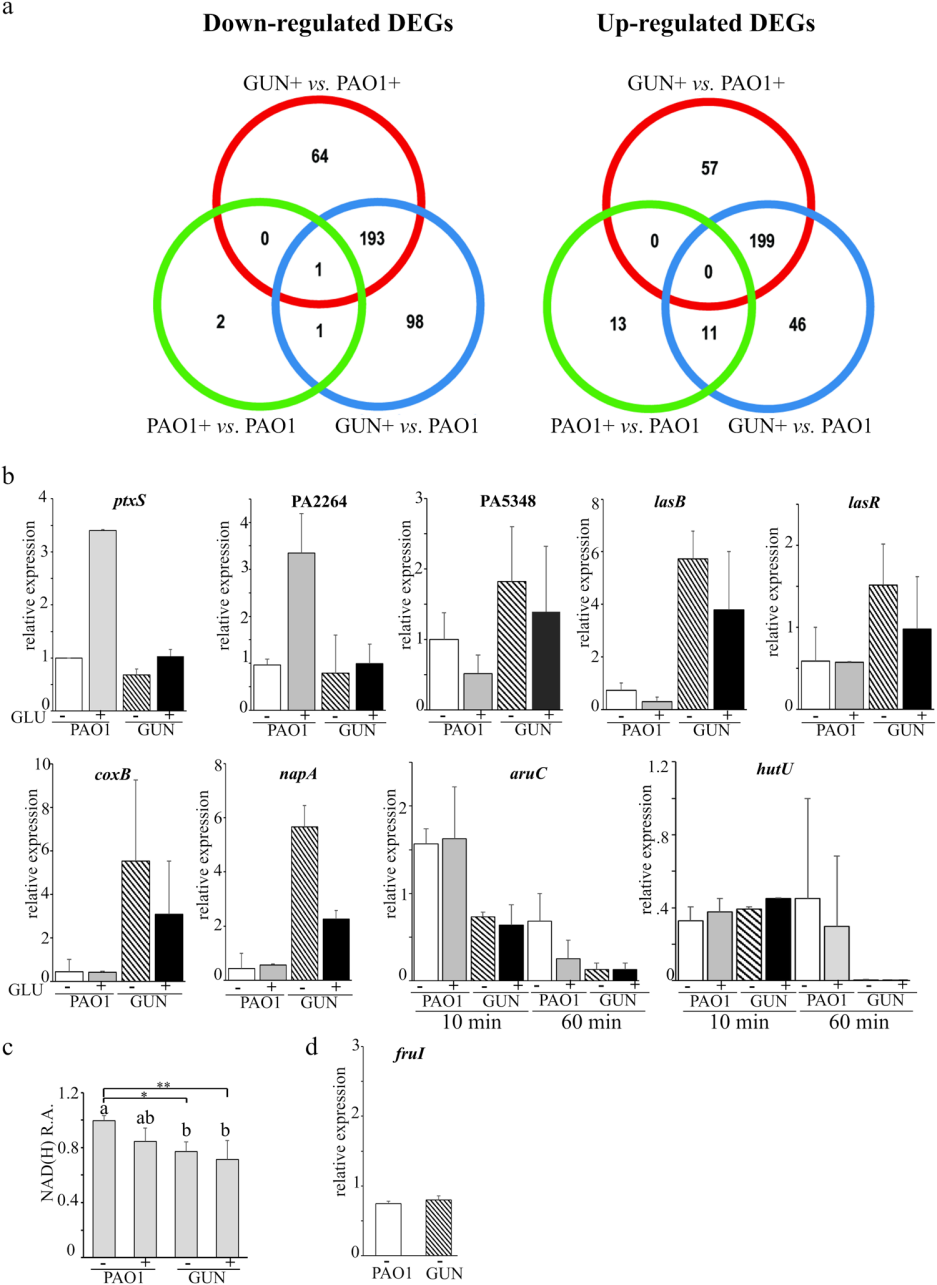
Transcriptome analysis results and differential gene expression validation. (**a**) Venn-diagrams of differentially expressed genes (DEGs). Venn diagrams representing the number of under- (left panel) and over-(right panel) expressed genes between GUN + and PAO1 + , GUN + and PAO1 and PAO1 and PAO1 + , as indicated. + , RNA extracted from cultures supplemented with glucose. (**b**,**d**). RT-qPCR mRNA analyses of differentially expressed genes. RNA was extracted from cultures of PAO1 or GUN mutant grown in M9-CAA up to OD_600_ = 0.4 and further incubated 60 min with ( + GLU) or without (- GLU) 0.4% (w/v) glucose and analysed by RT-qPCR with proper oligonucleotides. For *aruC* and *hutU* gene expression, RNA extracted 10 min after the addition of glucose was also analysed as indicated below the histograms. The bars represent relative expression with respect to the reference condition (i.e. PAO1 incubated 60 min without glucose; the replicate with lower expression was used for normalization). The lowest values obtained in two independent experiments, each performed on two technical replicates, are reported. The error bars represent the replicate range. (**c**). NAD(H) relative amount (R.A.). NAD(H) was extracted from cultures grown as described above and quantified as detailed in Supplementary Methods. Values were normalized for the lowest value obtained for PAO1 grown without glucose. Bars represent average (n = 4) with SD. Significance was evaluated with one-way Anova and Tukey post hoc test. *P < 0.05; **P < 0.01. Differences between means that share a letter (on top of the columns) are not statistically significant at 0.05 level.

To assess how the lack of glucose uptake systems may impact *P*. *aeruginosa* physiology, we analysed the global transcription profile of the GUN mutant by RNASeq in comparison with that of PAO1. RNA was extracted from GUN and PAO1 cultures grown in minimal medium with casamino acids as carbon source (a medium in which glucose is virtually absent, see Supplementary Fig. S2) up to mid log phase, and then incubated 60 min with 0.4% glucose. In these conditions, genes responding to the presence of glucose and its metabolism and possibly other genes not related to glucose metabolism, but responding to the lack of Glt, GntP and KguT, could be differentially expressed in GUN relative to PAO1. To discriminate between these two categories and identify glucose responsive genes in *P*. *aeruginosa*, RNASeq was performed also on RNA extracted from a culture of PAO1 grown in the same conditions as above, but without glucose addition, and the transcriptomes of PAO1 grown in the presence or absence of glucose were compared. Moreover, we compared the transcriptome of the GUN mutant grown in the presence of glucose with that of PAO1 grown either in the presence or in the absence of glucose. Notably, for the timespan of the experiment, PAO1 generation time did not significantly change upon glucose addition with respect to the culture without glucose (Supplementary Fig. S3), indicating that glucose is not required to sustain growth. Only 24 and 4 genes were up- and down-regulated (e.g. with a log_2_FC ≥ 1 or ≤ −1 and Padj < 0.05), respectively, upon glucose addition to PAO1 cultures (Fig. 3a). Down-regulated genes encoded components of amino acid or dipeptide ABC transporters, whereas the 24 up-regulated genes encoded functions mostly related to glucose catabolism (Fig. 3a and Supplementary Tables S2 and S3). Only 5 up-regulated genes were not directly related to glucose metabolism, namely *fruIKA*, encoding the phosphotransferase system for fructose transport, *gdhA* and *phuR*, coding for the glutamate dehydrogenase and the OM receptor of heme uptake, respectively. The *gntR* and *gltR* genes, encoding the GntR and GltR repressors of *gntP* and *glt-oprB* operons, respectively, were identified among the genes induced by glucose in PAO1. Conversely, the *ptxS* gene, which was previously demonstrated to be induced by glucose^5^, was not enclosed among differentially expressed genes because of its adjusted P value, which was slightly higher than the selected threshold (i. e. log_2_FC = 1.55; Padj = 0.08). However, RT-qPCR analysis of *ptxS* mRNA demonstrated that *ptxS* is actually up-regulated by glucose (Fig. 3b). On the whole, glucose effect on PAO1 transcriptome is similar to that observed in *P*. *putida*^2^, as in both cases, glucose addition results in the up-regulation of genes mainly implicated in glucose uptake and utilization and down-regulation of few genes controlling the transport and utilization of alternative carbon sources.

Although in our experimental settings glucose limitedly affected gene expression of PAO1 parental strain, the lack of glucose transporters in the GUN mutant had a striking impact on the transcriptome of the mutant relative to that of the wild type. 514 genes were differentially regulated at least two-fold in the GUN mutant with respect to PAO1 when both strains were grown in the presence of glucose. In detail, 258 and 256 genes were down- or up-regulated in the GUN mutant, respectively.

Genes coding for ribosomal proteins, for enzymes required for purine biosynthesis, for the RNA polymerase subunits (*rpoA*, *rpoB* and *rpoC*), and genes involved in energy metabolism like the NAD biosynthetic genes *nadD* and *nadE*, the *nuo* operon for NADH dehydrogenase and genes encoding cbb_3_−1 (*cco-1*), cbb_3_−2 (*cco2*) and cyo (*cyoAB*) terminal oxidases of the electron transport were enriched among genes down-regulated in the GUN mutant (Table 2; Supplementary Table S2). Conversely, genes for the subunits of aa_3_ terminal oxidase (i.e. *coxAB* and *coIII*), whose expression is induced upon nutrient starvation^23^, were up-regulated in the GUN mutant. Likewise, a subset of quorum sensing-dependent genes, including those involved in pyocyanin biosynthesis and efflux (namely *phz* and *mexGHI* genes^24^), lectins (*lecA* and *lecB*) and extracellular proteases (*lasA* and *lasB*), were more expressed in the GUN mutant relative to PAO1. Genes encoding chemotactic functions, pyoverdine biosynthetic genes and those encoding factors of the Apr and Xcp secretion systems were also up-regulated in the GUN mutant. Finally, some genes coding for global regulators (i.e. *dnr*, *argR*, *rhlR*, *hfq*, *gbdR*) were differentially expressed. In particular, *rpoD* and *rpoS*, encoding sigma factors σ^D^ and σ^S^, were down- and up-regulated in GUN, respectively (Table 3; Supplementary Tables S2 and S4). Interestingly, most of the above mentioned genes showed differential expression in the GUN mutant grown with glucose compared with PAO1 grown both with or without glucose (Table 3 and Supplementary Table S2).

**Table 2:** Enriched functional categories among DEGs.

GUN + *vs*. PAO1+	GUN + *vs*. PAO1
**Down-regulated DEGs** ^**a**^	
Ribosome (45)	Ribosome (39)
Purine metabolism (19)	Oxidative phosphorylation (20)
Carbon metabolism (24)	Purine metabolism (18)
RNA degradation (7)	Metabolic pathways (90)
Denitrification (7)	Arginine and proline metabolism (13)
Penthose phosphate cycle (8)	RNA degradation (6)
**Up-regulated DEGs** ^**a**^	
Quorum sensing (21)	Quorum sensing (16)
Phenazine biosynthesis (7)	Pyoverdine synthesis (6)
Xcp type II secretion system (5)	Apr type I secretion system (4)
Apr type I secretion system (5)	Phenazine biosynthesis (6)
Pyoverdine synthesis (6)	Xcp type II secretion system (5)
Bacterial chemotaxis (10)	Nitrogen metabolism (9)
AMB^b^ biosynthesis (3)	Bacterial chemotaxis (8)

^a^Categories are ordered according to increased adjusted probability values. The number of DEGs belonging to each category is indicated in brackets.

^b^AMB, L-2-amino-4-methoxy-trans-3-butenoic acid.

**Table 3:** Differential expression of selected *P*. *aeruginosa* genes.

Locus^a^	Name	Fold Change			Description
		PAO1 + *vs*. PAO1−	GUN + *vs*. PAO1+	GUN + *vs*. PAO1−	
***Genes up-regulated in GUN mutant*** ^***b***^					
**PA0105**	*coxB*	0.8	8.2	6.5	cytochrome C oxidase subunit
**PA1174**	*napA*	0.8	4.8	3.6	nitrate reductase subunit
**PA1430**	*lasR*	1.0	1.7	1.8	transcriptional regulator LasR
PA2426	*pvdS*	2.5	1.9	4.8	sigma factor PvdS
PA2570	*lecA*	0.7	4.7	3.1	LecA lectin
PA3361	*lecB*	0.6	5.2	2.9	fucose-binding lectin PA-IIL
PA3477	*rhlR*	0.8	2.4	1.9	transcriptional regulator RhlR
PA3478	*rhlB*	0.8	3.0	2.3	rhamnosyltransferase subunit B
PA3479	*rhlA*	0.7	3.3	2.5	rhamnosyltransferase subunit A
PA3622	*rpoS*	0.9	2.6	2.4	sigma factor σ^S^
**PA3724**	*lasB*	0.5	7.8	3.5	elastase LasB
PA4210	*phzA1*	0.4	6.2	2.7	phenazine biosynthesis protein
PA4211	*phzB1*	0.5	7.2	3.2	phenazine biosynthesis protein
**PA5348**	PA5348	0.8	3.0	2.4	probable DNA-binding protein
***Genes down-regulated in the GUN mutant*** ^***b***^					
PA0576	*rpoD*	1.1	0.4	0.5	sigma factor σ^D^
PA0893	*argR*	0.6	0.6	0.4	transcriptional regulator ArgR
**PA0895**	*aruC*	0.5	0.3	0.2	acetylornithine aminotransferase
**PA2259**	*ptxS*	2.9	0.5	1.4	transcriptional regulator PtxS
**PA2264**	PA2264	3.4	0.5	1.5	conserved hypothetical protein^c^
PA2320	*gntR*	3.7	0.3	1.1	GntR transcriptional regulator
PA4006	*nadD*	0.9	0.5	0.5	NAM adenylyltransferase
PA4920	*nadE*	1.5	0.5	0.8	NAD synthetase
**PA5100**	*hutU*	0.8	0.0	0.0	urocanate hydratase
PA5105	*hutC*	0.8	0.2	0.1	Transcriptional regulator HutC
***Genes for glucose IM transporters deleted in this work***					
PA2262	*kguT*	1.3	0.3	0.4	2-ketogluconate transporter
PA2322	*gntP*	35.5	0.0	0.6	gluconate permease
PA3187	*gltK*	52.0	0.1	6.7	ABC transporter ATP-binding protein
PA3188	*gltG*	31.6	0.0	0.4	sugar ABC transporter permease
PA3189	*gltF*	3.5	0.3	0.9	probable permease of ABC sugar transporter

^a^Boldface characters, genes analysed by RT-qPCR.

^b^Genes up- and down-regulated in GUN mutant with respect to PAO1 (with and/or without glucose).

^c^The protein contains a domain belonging to the Gluconate 2-dehydrogenase subunit 3 family (InterPro IPR027056).

The RNASeq data for a subset of differentially expressed genes (Table 3) were validated by RT-qPCR and/or phenotypic assays. As for the RT-qPCR analyses, the RNA was extracted from PAO1 and GUN cultures grown in the same conditions as the cultures for RNASeq. The RNA of the GUN mutant grown in the absence of glucose was also enclosed in the analysis. We tested genes with different expression patterns, namely PA2264, which was found by RNASeq to be up-regulated in PAO1 upon glucose addition, PA5348, *lasB*, *coxB* and *napA*, all up-regulated in GUN with respect to PAO1, and the two genes *aruC* and *hutU*, which are down-regulated in the GUN mutant. Since *lasB* gene is positively regulated by LasR^25^, we also analysed the transcription of *lasR*, which was not included among differentially expressed genes because its log_2_FC was slightly below the selected threshold (log_2_FC = 0.9).

Overall, the RT-qPCR results (Fig. 3b) were consistent with those of the RNASeq experiment (Table 3). For most genes, the transcription profile of the GUN mutant was not affected by glucose. The only exceptions were *coxB* and *napA* (coding for subunits of the aa_3_ terminal oxidase and of the periplasmic nitrate reductase, respectively), whose expression, although remaining much higher than in PAO1, was reduced in the GUN mutant upon glucose addition. The RT-qPCR results confirmed the strong repression of *hutU* and *aruC* genes of the assimilative pathways of histidine and arginine in the GUN mutant in comparison to PAO1 without glucose. This observation was unexpected because both PAO1 and the GUN mutant can rely only on casamino acids as carbon source in the absence of glucose. Thus, in principle, they should have a similar expression profile of genes involved in amino acids transport and metabolism. Indeed, both genes were expressed in all tested strains/conditions in cultures at an early time point (10 min after the addition of glucose; Fig. 3b).

Finally, since *nadD* and *nadE* were down-regulated in the GUN mutant with respect to PAO1 (Table 3), we measured total NAD(H) in PAO1 and GUN. In agreement with transcriptomic data, we found that in the GUN mutant growing with or without glucose, NAD(H) cellular content was significantly reduced in comparison to PAO1 growing in the absence of glucose (Fig. 3c), whereas the difference was not significant with respect to PAO1 growing with glucose.

Overall, these results show that the inactivation of glucose uptake systems largely affects *P*. *aeruginosa* gene expression regardless of the presence of glucose.

### Quorum sensing signal molecules and biofilm formation are altered in the GUN mutant

**Figure 4:**
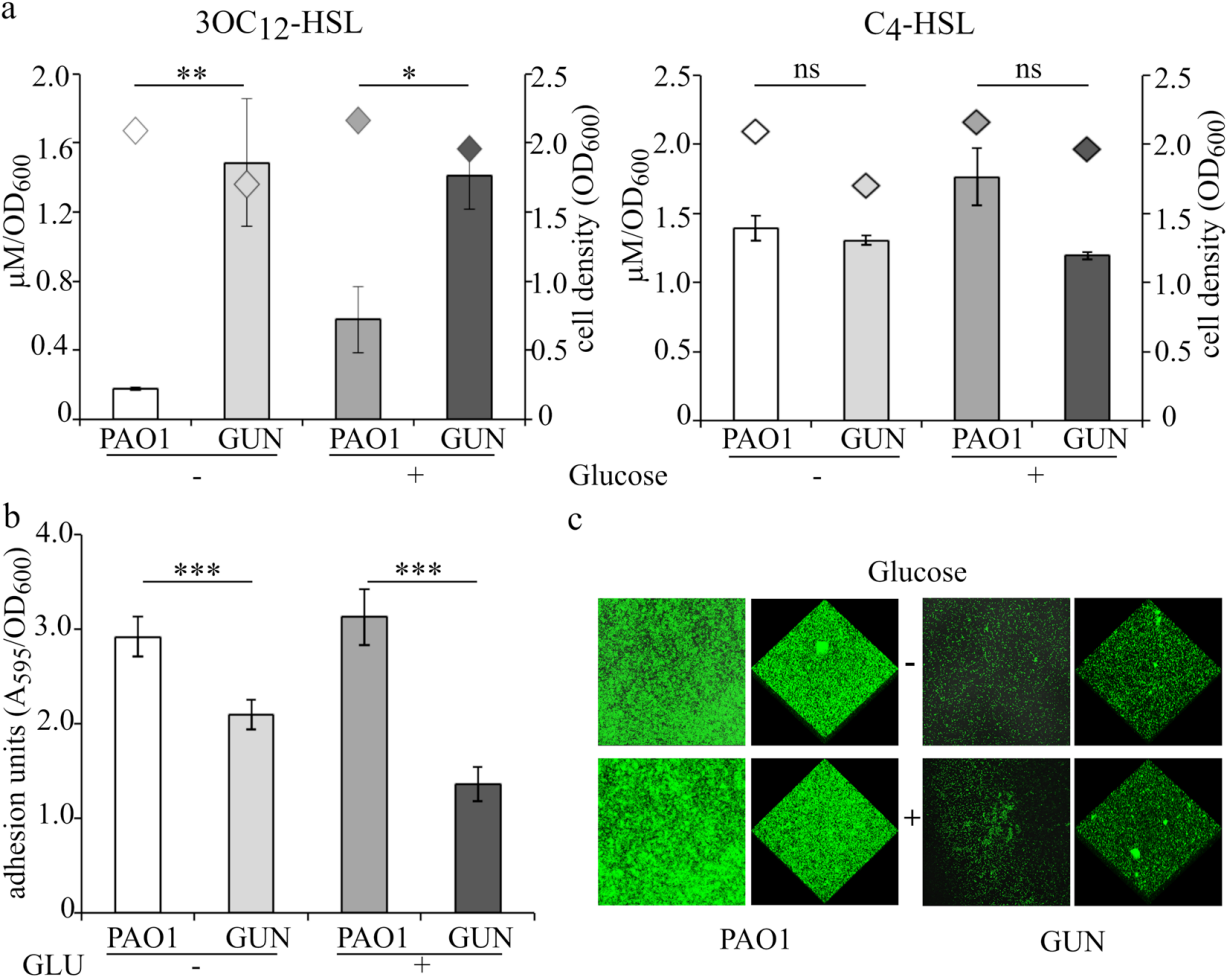
Autoinducers production and adhesion assays. (**a**) Histograms reporting the maximal production of QS signal molecules 3OC_12_-HSL and C_4_-HSL normalized to the cell density of the culture in the indicated strains grown in M9-CAA in the absence (−) or in the presence (+) of 0.4% (w/v) glucose; diamonds indicate the corresponding cell density (OD_600_). Median values of three independent experiments are reported with SD (*P < 0.05; **P < 0.01; ns, not statistically significant). (**b**) Adhesion units determined as A_595_ of the crystal violet stained solubilized biofilms formed by the indicated strains in M9-CAA in the absence (−) or in the presence (+) of 0.4% (w/v) glucose, normalized to the cell density (OD_600_) of the corresponding planktonic cultures. Median values of three independent experiments, each performed on 6 technical replicates, are reported with SD (***P < 0.001). (**c**) Representative LSCM images of biofilms formed by the indicated strains constitutively expressing GFP grown in M9-CAA in the absence (−) or in the presence (+) of 0.4% (w/v) glucose.

Transcriptomic analysis and/or RT-qPCR showed a significant up-regulation of QS-dependent genes in the GUN mutant. Accordingly, the QS receptor genes *lasR* and *rhlR*, responding to *N*−3-oxododecanoyl-homoserine lactone (3OC_12_-HSL) and *N*-butanoyl-homoserine lactone (C_4_-HSL) signal molecules, respectively, were also up-regulated in the GUN mutant (Table 3). To test whether the *las* and *rhl* QS circuits were actually altered in the GUN mutant, the levels of 3OC_12_-HSL and C_4_-HSL signals were measured in PAO1 and GUN cultures grown in minimal medium supplemented with casamino acids, with or without glucose. As shown in Fig. 4a, maximal 3OC_12_-HSL production was significantly increased in the GUN mutant relative to PAO1. This effect did not depend on the differential ability of the two strains to import glucose, since it occurred also in the absence of glucose. Conversely, C_4_-HSL levels were not significantly affected in all the tested conditions.

Biofilm formation in *P*. *aeruginosa* is a pleiotropic phenotype depending also upon QS and involving lectins and rhamnolipids, which expression was up-regulated in the GUN mutant (i.e. *lecA*, *lecB* and *rhlAB* genes; Table 3). Hence the biofilm forming ability of the GUN mutant was investigated by means of both standard crystal violet assay in polystyrene 96-well microtiter and laser scanning confocal microscopy (LSCM) on glass slides (Fig. 4b,c). These experiments revealed that biofilm formation was reduced in the GUN mutant relative to PAO1 irrespective of glucose presence in the medium.

### Virulence-related traits of *P*. *aeruginosa* mutants defective in glucose uptake

**Figure 5:**
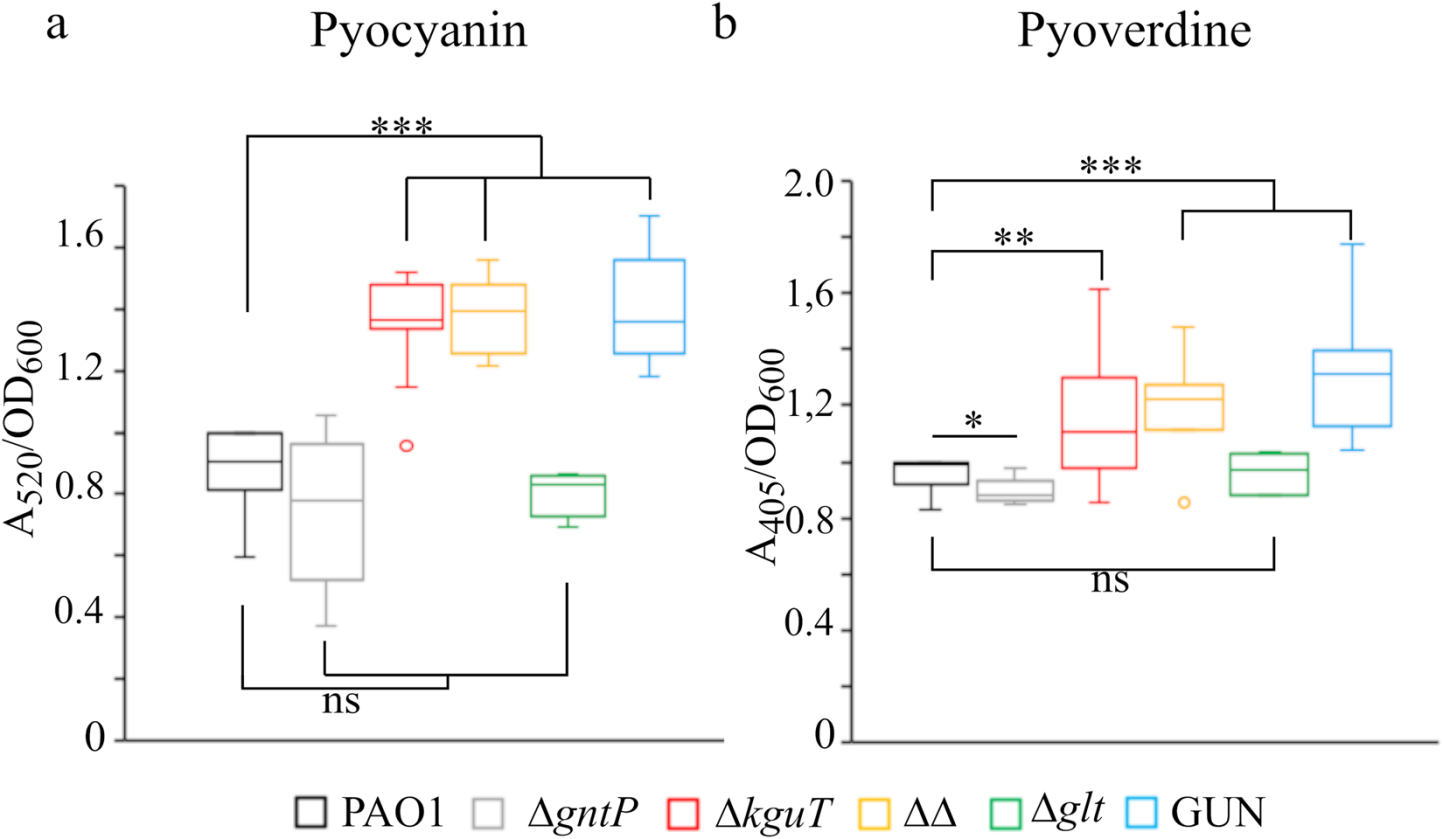
Pyocyanin and pyoverdine production by glucose uptake mutants. Cultures of the strains listed above the panels were grown 24 h at 37 °C in LD. ΔΔ indicates PAO1 Δ*gntP* Δ*kguT* strain. Pyocyanin (**a**) and pyoverdine (**b**) were measured as described in Supplementary Experimental procedures. The median (line) is reported inside the boxes (n ≥ 5). The whiskers represent the minimum and maximum values observed. Significance was estimated with one-way Anova and Tukey post-hoc analysis (ns, not significant; *P < 0.05; **P < 0.01; ***P < 0.001), only the results relative to PAO1 are reported.

The glucose uptake defective mutants were assayed for selected virulence traits, according to the results of GUN transcriptomic analysis and/or to literature data linking glucose and *P*. *aeruginosa* pathogenic potential^20,26–28^. In particular, we investigated the production of: extracellular proteases; pyocyanin, a blue redox-active molecule inducing oxidative stress in host cells; pyoverdine, a siderophore allowing iron acquisition and bacterial growth within the host; rhamnolipids, biosurfactants playing a role in biofilm formation and chronic infection; growth in microaerophilic and in anaerobic environments. We found that all strains carrying the *kguT* deletion produced more pyocyanin, pyoverdine and rhamnolipids than PAO1, whereas the *glt* deletion had no effect on their production. Single *gntP* deletion slightly increased and reduced rhamnolipids and pyoverdine production, respectively, and had no effect on pyocyanin (Fig. 5 and Supplementary Fig. S4). It should be noted that, albeit statistically significant, the differences between the mutants and PAO1 in the production of both pyoverdine and rhamnolipids were small and thus their biological meaning uncertain. As for extracellular proteases, they were comparably produced by all strains in the absence of glucose. Glucose supplementation repressed protease production by PAO1 and by the single mutants, whereas it did not affect production by the Δ*gntP* Δ*kguT* and GUN mutants. Finally, none of the tested mutations affected the growth in microaerophilic or anaerobic conditions (Supplementary Fig. S4).

### Interfering with oxidative route of glucose import has different outcomes on the virulence in *Caenorhabditis elegans* and *Galleria mellonella* infection models

The virulence of the glucose uptake defective mutants was assayed in two *P*. *aeruginosa* infection models, i.e. the nematode *Caenorhabditis elegans* and the *Galleria mellonella* insect larvae. It should be noted that the paths of *P*. *aeruginosa* invasion, and presumably the physiology of bacterial cells in the two systems, are largely different. In particular, while in *G*. *mellonella* infections bacteria are injected in the insect hemolymph, thus causing an acute infection that kills the larvae within 18–40 h at 37 °C, in *C*. *elegans* slow-killing assay worms are fed on *P*. *aeruginosa* cells, which colonize the intestine while staying embedded in an extracellular matrix and kill the worms over the course of several days at 20 °C^29–32^.

In *C*. *elegans* slow-killing assay (Supplementary Fig. S5), lethality was visible 7–8 days post-infection with PAO1. The Δ*kguT* and the Δ*gntP* Δ*kguT* mutants were slightly but significantly more virulent than PAO1, whereas Δ*gntP*, Δ*glt* and GUN mutants did not show significant differences in virulence relative to the wild type.

**Figure 6:**
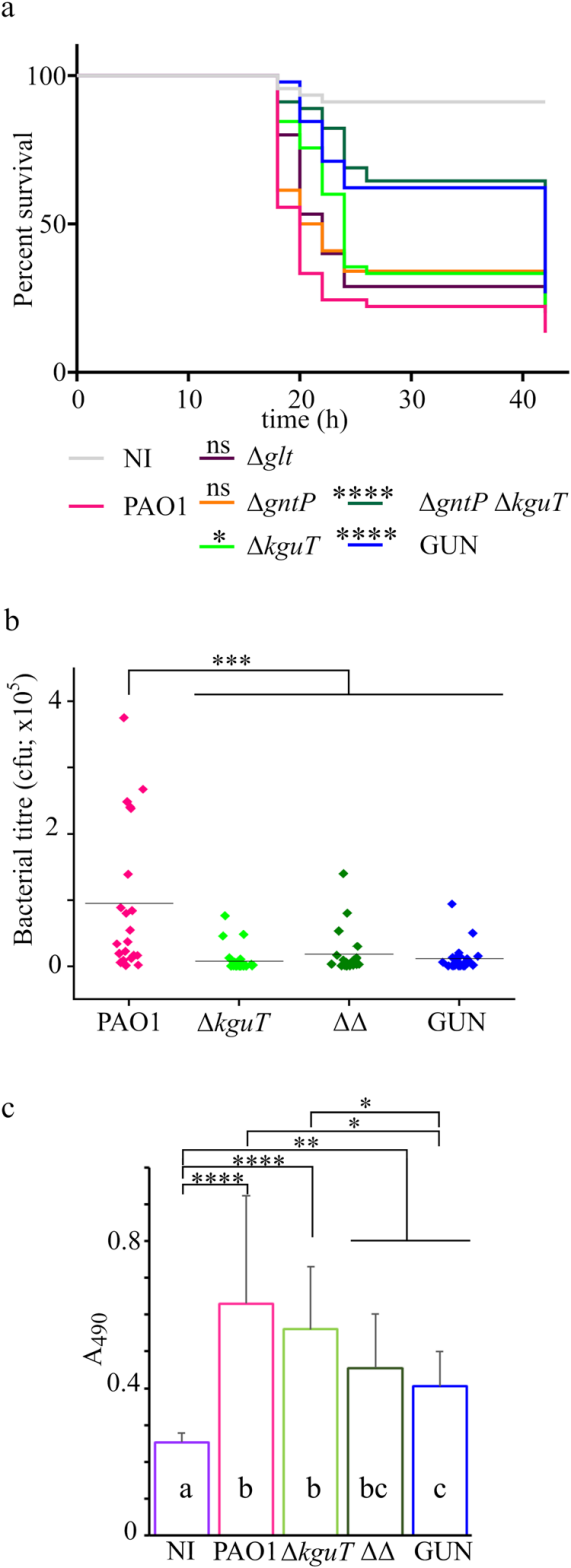
*G*. *mellonella* infection assay. (**a**) Survival curves of *G*. *mellonella* larvae infected with glucose uptake mutants. Kaplan-Meyer curves represent results deriving from three independent experiments in which groups of 15 larvae were injected with the indicated strains. The average number of *P*. *aeruginosa* cells injected was 26 ± 5 (PAO1), 31 ± 6 (Δ*glt*), 30 ± 5 (Δ*gntP*), 28 ± 8 (Δ*kguT*), 27 ± 6 (Δ*gntP* Δ*kguT*), 34 ± 6 (GUN). h, hours post-infection. NI (not infected), larvae injected with sterile physiological solution. Significance was estimated with log-rank test; only the results relative to PAO1 are reported (*P < 0.05; ****P < 0.0001; ns, not significant). (**b**) The dots represent bacterial titre 16 h post-infection in the hemolymph of larvae (n = 24 ± 4) infected with 10 µl of physiological solution containing the following number of bacterial cells: 33 ± 8 (PAO1), 37 ± 5 (Δ*kguT*), 34 ± 8 (ΔΔ, Δ*gntP* Δ*kguT*), 34 ± 9 (GUN). The results were plotted with OriginPro (OriginLab). The line represents the mean value. Significance was estimated with one-way Anova and Tukey post-hoc analysis (***P < 0.001). Differences among the Δ*kguT*, Δ*gntP* Δ*kguT* and GUN mutants were not significant at 0.05 level. (**c**) Phenoloxidase activity assay. Hemolymph was collected 4 h post-infection with 20000 cfu of the bacterial strains listed in the panel. The hemolymph samples deriving from groups of ten larvae infected with the same strain were pooled and phenoloxidase activity at 30 min was estimated as described in Methods. The results of four experiments done in different days (n = 12) are shown. Bars represent average with SD. Significance was estimated with one-way Anova and Tukey post-hoc analysis (****P < 0.0001; **P < 0.01; P*P < 0.05). Differences between means that share a letter (on the columns) are not statistically significant at 0.05 level.

Preliminarily to *G*. *mellonella* infection with *P*. *aeruginosa*, we measured the concentration of glucose in the larvae hemolymph. We observed that glucose was relatively scarce (mean value 11.2 μM, corresponding to 2.0 μg/ml; Supplementary Fig. S2) with respect to a previous estimation^33^, probably because the larvae were starved for 24h-48h before starting the experiment. In the larva infection model, the Δ*kguT* mutant was less virulent than PAO1, and significant attenuation was shown by the Δ*gntT* Δ*kguT* and GUN multiple mutants. Indeed, 24 h post-infection (h.p.i.) the mortality of larvae injected with either Δ*gntT* Δ*kguT* or GUN was lower than 40%, while the mortality rate of larvae challenged with wild type PAO1 exceeded 75% (Fig. 6a). To test whether attenuation of strains lacking the *kguT* gene was due to slower growth of the mutants *in vivo*, the hemolymph of larvae infected with a comparable number (between 20 and 30) of PAO1, Δ*kguT*, Δ*gntT* Δ*kguT* or GUN cells was recovered 16 h.p.i. and bacteria were titrated. As shown in Fig. 6b, the *in larva* bacterial load was significantly lower for all tested mutants compared to PAO1, whereas no significant difference was detectable among the mutants.

We also evaluated the stimulation of the larvae immune system by measuring the activation of prophenoloxidase cascade in the hemolymph^34^ after larvae infection with a lethal dose of PAO1 or of single and multiple mutants lacking *kguT* (Fig. 6c). As expected, we observed an increase in the phenoloxidase production in larvae infected with either PAO1 or any of the mutants with respect to larvae injected with sterile physiological solution. Phenoloxidase was significantly less abundant in larvae infected with the Δ*gntT* Δ*kguT* or GUN mutants with respect to those infected with PAO1 or the Δ*kguT* single mutant.

## Discussion

In this work we describe the generation and characterization of a collection of single and multiple mutants of *P*. *aeruginosa* PAO1 with unmarked, non-polar deletions (see Supplementary results and Supplementary Fig. S6 for the analysis of mutation polarity) of genes encoding IM proteins involved in glucose transport. The inability of the GUN mutant to grow on glucose as unique carbon source confirms that *P*. *aeruginosa* does not have any other IM transporter for this sugar besides Glt, GntP and KguT. Similarly, the failure of the double mutant Δ*gntP* Δ*kguT* and of the single mutant Δ*kguT* to grow on gluconate and on 2-KG, respectively, confirms that GntP and KguT are the only transporters involved in the uptake of intermediates of the oxidative branch of glucose utilization, with the latter being specifically required for 2-KG uptake. Conversely, the lack of any apparent growth defect on minimal medium supplemented with glucose (or gluconate) of strains defective for the glucose-specific OprB porin and for its paralogue OprB2, whose expression is also regulated by glucose, shows that glucose and gluconate can cross the OM through other porins. Indeed, the amino acid- and imipenem- specific OprD porin has been shown to facilitate gluconate entry in *P*. *aeruginosa*^35^. Gill *et al*.^36^ observed slower growth in minimal medium supplemented with glucose by a PAO1 Δ*oprB* mutant. This discrepancy with respect to our results could be explained by the known heterogeneity of PAO1 sublines^37^ used in different laboratories, and may be due to differential expression of genes encoding facilitators of glucose entry in the various PAO1 sublines.

The transcription profile of genes involved in glucose, gluconate and 2-KG transport was coherent with their expected regulation by GltR, GntR and PtxS, respectively^1^. Repression by GntR and PtxS is relieved only if their cognate effectors (i.e. gluconate/6-phosphogluconate for GntR and 2-KG for PtxS) are present within the cell cytoplasm. Conversely, phosphorylation of the response regulator GltR, which determines its detachment from DNA, is triggered by 2-KG interaction with the histidine kinase GtrS in the periplasm^1^. Consistently, in the presence of glucose, the GltR-regulated *glt-oprB* operon was highly and comparably expressed in PAO1 and in the GUN mutant, whereas the expression of the *gntP* and *kgu* operons was lower in the mutant and similar to that observed in PAO1 grown in the absence of glucose. The same expression pattern was shown by other genes connected with glucose catabolism and regulated by HexR, whose effector is synthesized only upon glucose entry into the cell^1^.

We observed enhanced transcription of both the *gntR*, *gltR* and *ptxS* repressor genes and their *gntP*, *glt-oprB* and *kgu* target operons upon glucose addition to PAO1 cultures. This result can be explained considering that i) transcription of *gntR*, *gltR* and *ptxS* genes is subject to negative auto-regulation and, limitedly to *gltR*, also to repression by HexR; and ii) increased intracellular concentration of GntR, GltR and PtxS should not result in repression of their target operons as long as glucose is present because of the allosteric induction of GntR and PtxS and the GltS-dependent phosphorylation of GltR triggered by glucose derivatives^1^. We speculate that increasing the GntR, GltR and PtxS amount as part of the response to glucose may contribute to quickly switch off the transcription of the operons regulated by such repressors upon glucose exhaustion.

The effect of glucose addition on PAO1 transcriptome was limited to the induction of 24 genes, mostly involved in glucose transport and catabolism, and to the down-regulation of few genes mainly belonging to ArgR regulon connected with the transport and utilization of arginine^38^, which is likely used by PAO1 as carbon source when growing in minimal medium with casamino acids. Glucose-dependent induction of *fruIKA* in PAO1 and in the GUN mutant (Supplementary Table S2 and Fig. 3d) was likely due to contaminating fructose originated by spontaneous glucose isomerization to fructose^39^.

The lack of glucose transporters deeply impacts *P*. *aeruginosa* transcriptome, with a large numbers of genes belonging to various pathways differentially regulated in the GUN mutant with respect to PAO1. The results of RT-qPCR and phenotypic assays were consistent with those of the RNASeq analysis and showed that the GUN mutant is endowed with a complex phenotype having decreased NAD(H) content, dysregulated quorum sensing, reduced biofilm formation and virulence attenuation in *G*. *mellonella* in spite of enhanced production of virulence factors like pyocyanin, pyoverdine and extracellular proteases. The panorama of genes differentially expressed in GUN provides some hints on the physiology of this mutant strain. Downregulation of genes typically expressed in the exponential phase and connected with growth, like those encoding ribosomal proteins or the RNA polymerase subunits, together with the up-regulation of *rpo*S, encoding the alternative sigma factor σ^S^, testifies the stress condition of the GUN strain. Moreover, *aru* and *hut* genes for arginine and histidine utilization are equally expressed in PAO1 and GUN mutant in cultures in mid exponential phase (i.e. 10 min after glucose addition to cultures at OD_600_ = 0.4), whereas they are down-regulated in the GUN mutant relative to PAO1 at a later time point, suggesting that PAO1 and the GUN mutant could use the amino acids in the medium in a different order and/or at a different rate. *aru* and *hut* operons are regulated by ArgR and HutC, whose genes are down-regulated in the GUN mutant (Table 3), and by the CbrA/CbrB response regulator, which activates transcription of the arginine and histidine utilization operons in response to an unknown signal^40^.

The iron starvation sigma factor *pvdS*, a major global regulator of iron uptake systems and virulence^41^, is up-regulated in the GUN mutant. The consequent up-regulation of PvdS target genes (e.g. *pvd* pyoverdine biosynthetic genes) and the enhanced pyoverdine production may be linked to a dysregulated iron metabolism. Indeed, transcriptomic data suggest that in the GUN mutant a complex reorganization of the electron transport chain may take place, and this would require the synthesis of heme- (and thus iron-) containing proteins, possibly enhancing the iron demand of the mutant. However, a regulatory link coordinating iron supply and carbon metabolism has been recently established in pseudomonads^42,43^, opening the possibility that *pvdS* up-regulation may be actually part of a stress response elicited by carbon metabolism defects of the GUN mutant.

It is likely that the altered QS cascade and biofilm formation observed in in the GUN mutant may also be related to its complex metabolic dysregulation. We observed increased production of the 3OC_12_-HSL signal molecule in the GUN mutant relative to PAO1, in line with increased RNA level of the *lasR* gene and with the enhanced production of LasR-controlled virulence factors, like pyocyanin and proteases. The *las* QS system usually exerts a positive regulatory role on the C_4_- HSL-dependent *rhl* QS circuit. Indeed, the *rhlR* gene and the RhlR-dependent *rhlAB* genes for rhamnolipids production were up-regulated, but unexpectedly production of the C_4_-HSL signal molecule was unaffected. QS circuits in *P*. *aeruginosa* not only respond to cell density, since their activation is finely modulated by a plethora of environmental and metabolic stimuli^44,45^. Moreover, hierarchical organization of the QS cascade in *P*. *aeruginosa*, with the *las* system being required for full activation of the *rhl* QS circuit, has been mainly described in rich media, while this connection is possibly missing or altered in other growth conditions^46,47^. Altered QS cascade may in turn interfere with biofilm formation, as it is known that a large number of factors controlled by QS may impact biofilm formation^48^. However, biofilm formation is a pleiotropic phenotype affected by multiple environmental and metabolic stimuli whose signalling pathways are altered in the GUN mutant, including QS, iron uptake systems, and arginine metabolism^49,50^. Hence, while altered biofilm formation in the GUN mutant relative to PAO1 is not surprising, defining the specific impact of GUN-controlled phenotypes on biofilm formation is a puzzling issue that will deserve further investigation.

Interestingly, the global transcription profile and most of the assayed phenotypic traits differ between the GUN mutant and PAO1 irrespective of the presence of glucose. In particular, the GUN mutant seems to perceive nutrient starvation in conditions in which PAO1 does not. As an example, in the GUN mutant, a strong up-regulation of *cox* genes encoding the aa3 terminal oxidase, which is expressed in nutrient starvation conditions^23^, is observed (Table 3 and Fig. 3b). This suggests that the investigated glucose uptake systems may have other functions independent of glucose transport. It is tempting to speculate that one or more of the proteins missing in the GUN mutant may participate in the regulation of cell metabolism, for instance by modulating the activity of sensor histidine kinases (HK) of two-component systems as it has been found for the *E*. *coli* dicarboxylic acid transporters DauA, which regulates the HK DcuS^51^. KguT could be a good candidate for such an accessory function as it plays a major role in determining relevant phenotypes *in vitro* and *in vivo*. Indeed, we observed enhanced *in vitro* secretion of the virulence factors pyocyanin and pyoverdine by all mutants lacking the KguT transporter. On the other hand, such mutants showed attenuated virulence in *G*. *mellonella*, suggesting that pyocyanin and pyoverdine increased production, if occurring also *in vivo*, is either scarcely relevant or counteracted by other mutants’ features detrimental to the *in larva* growth.

Larvae infected with the GUN mutant showed lower phenoloxidase production than those infected with PAO1 or the Δ*kguT* single mutant. Pro-phenoloxidase cascade is triggered not only by the lipopolysaccharide of the bacterial OM, but also by specific bacterial proteins like thermolysin^52^. Further analyses will be required to assess whether factors stimulating pro-phenoloxidase cascade are differentially produced by the GUN mutant with respect to PAO1 and the Δ*kguT* strain.

The single Δ*kguT* and the double Δ*gntP* Δ*kguT* mutants were slightly more virulent than PAO1 in *C*. *elegans*. Thus, the bacterial ability to import (and use) glucose, as well as the accessory function of the glucose uptake transporters in the regulation of cell metabolism highlighted in this study, may have a different relevance in different infection models. In agreement with this observation, PAO1 mutants defective in glucose metabolism genes (i.e. Δ*oprB*, Δ*gltK*, Δ*gtrS* and Δ*glk* mutants) were shown to cause a reduced bacterial load with respect to wild type PAO1 in the airways of hyperglycaemic mice, but not in those of normal ones^36^. This suggests that targeting glucose metabolism with specific drugs may differentially impact the outcome of chronic and acute human infections caused by *P*. *aeruginosa*, depending on the particular nutritional milieu at the site of infection and on the specific strategy adopted by *P*. *aeruginosa* to thrive within the host in each case. A deeper knowledge of bacterial physiological state in the different human infections together with the availability of proper preclinical infection models recapitulating them are needed to develop effective therapeutic strategies based on the inhibition of bacterial metabolic functions.

## Methods

### Bacteria, plasmids and oligonucleotides

Bacterial strains, plasmids and oligonucleotides used in this study are listed in Supplementary Table S1. *P*. *aeruginosa* genome coordinates throughout this work refer to PAO1 strain, Genbank Accession Number NC_002516.2. Construction of PAO1 deletion mutants in glucose uptake genes by gene replacement^53^ and *kguT* cloning in pGM2071 plasmid are detailed in Supplementary Methods. Bacterial cultures were grown in LD broth or M8 and M9 minimal media^54,55^. M9-CAA is M9 supplemented with 0.2% (w/v) casamino acids (i.e. Casein Hydrolysate, Sigma-Aldrich). When needed, media were supplemented as follows: 100 µg/ml ampicillin, 150–300 µg/ml carbenicillin, 10 µg/ml nalidixic acid, 0.4% (w/v) glucose, 0.4% (w/v) gluconate, 0.1% (w/v) 2-ketogluconate, 0.5–1% (w/v) succinate, 0.02% (w/v) arabinose, 10% (w/v) sucrose and 0.05% (v/v) Triton X-100. To prevent calcium precipitation, a calcium free solution of 2-ketogluconate was prepared as previously indicated^5^.

### RNA extraction for RNA-Seq

Total RNA was extracted as previously described^56^ from *P*. *aeruginosa* cultures grown in M9-CAA at 37 °C up to OD_600_ = 0.4 and further incubated 60 min with or without 0.4% (w/v) glucose; biological duplicates of the experiments were performed. Ribosomal RNA was depleted from 1 µg of total RNA with the RiboZero Gram positive kit (Illumina) according to the manufacturer’s instructions. Strand specific RNA-Seq libraries were prepared with the ScriptSeqTM v2 RNAseq library preparation kit (Illumina) from 50 ng of rRNA-depleted RNA. The libraries were sequenced on a MiSeq Illumina sequencer; 75 bp Single End reads were produced.

### RNA-Seq data analysis and PAO1 genome annotation

The strategy that we followed to obtain a comprehensive annotation of PAO1 genome is detailed in Supplementary Methods. Bowtie 2 (v2.2.6)^57^ was used to align raw reads to *P*. *aeruginosa* PAO1 genome (GCF_000006765.1) and only high quality reads (MAPQ > 30) were considered for the subsequent step of the analysis. The R^58^ package DESeq. 2 (v1.14.1)^59^ was used to normalize the counts and to produce the differentially expressed gene lists, setting independent filtering to FALSE. The enrichment of functional categories was calculated using Fisher test and Benjamini Hochberg correction for multiple testing. The categories with an adjusted P value (Padj) ≤ 0.05 were considered significantly enriched.

### RT-qPCR mRNA analysis

RT-qPCR (Reverse Transcription-quantitative PCR) was performed on 1 µg of RNA extracted from two independent cultures for each strain grown as for the RNASeq. Technical details and the list of primers specific for each analysed gene is provided in Supplementary Methods and Table S1. Two technical duplicates were performed for each biological replicate. 16 S rRNA was used as reference gene.

### *In vitro* phenotypic assays

Anaerobic and microaerophilic growth condition and a detailed description of the *in vitro* phenotypic assays applied in this work are provided in Supplementary Methods. In brief, NAD(H) content in samples of PAO1 and GUN cultures grown as for the RNASeq was quantified by a cyclic assay^60^. Pyocyanin, pyoverdine and rhamnolipids production was estimated as described^54,61,62^ on stationary cultures grown in LD at 37 °C. Extracellular proteases were tested by spotting supernatants of cultures grown 17 h at 37 °C in M9-CAA with or without 0.4% glucose onto casein-agar plates. Levels of QS signal molecules in *P*. *aeruginosa* PAO1 wild type and GUN mutant culture supernatants were determined at different times during bacterial growth in M9-CAA with or without 0.4% (w/v) glucose as described^63^. Maximal QS signal molecule concentration determined during bacterial growth is reported in Fig. 4a. Biofilm formation by PAO1 wild type GUN mutant grown in M9-CAA with or without 0.4% (w/v) glucose for overnight (i.e. 16 h) was assessed using the microtiter plate biofilm assay^64^. For microscopic visualization of biofilm, *P*. *aeruginosa* PAO1 wild type and GUN mutant constitutively expressing GFP *via* the pMRP9–1 plasmid^65^ were grown in an 8-well chamber slide, as previously described^66^, in M9-CAA in the absence or in the presence of 0.4% (w/v) glucose. Biofilms formation was examined after 3 days incubation by using the Leica TCS SP5 confocal microscope. For QS signal molecules and CV biofilm quantification statistical analyses were performed by using the GraphPad Prism software (San Diego, CA, USA), with a Tuckey test of the One-way ANOVA tool.

### *C*. *elegans* slow killing assay

50 worms synchronised at the L4 stage^67,68^ (see Supplementary Methods) were individually transferred from a Nematode Growth Medium (NGM) plate (US Biological) onto an NGM plate supplemented with 5-Fluoro-2′-deoxyuridine thymidylate synthase inhibitor (FUDR, Sigma-Aldrich), an inhibitor of DNA synthesis preventing *C*. *elegans* reproduction, without interfering with nematodes development and ageing^69^. Each plate was seeded with one bacterial strain using a sterilized platinum wire with the aid of a stereomicroscope. As negative control, 50 synchronized worms were transferred to an NGM plus FUDR plate seeded with *E. coli* OP50. Plates were incubated at 20 °C and the number of live nematodes was counted every day over a period of 21 days. A worm was considered dead when it no longer responded to touch. The experiment was repeated at least three times for each strains. The software GraphPad Prism (GraphPad Software, San Diego, CA, USA) was used to plot charts and perform statistical analyses.

### *G*. *mellonella* infection experiments

*P*. *aeruginosa* cultures for *G*. *mellonella* infections were grown to mid-log phase in LD medium, washed, re-suspended to OD_600_ = 1 and diluted in physiological solution (0.9% (w/v) NaCl) accordingly to the required inoculum size. *G*. *mellonella* caterpillars in the final instar larval stage were purchased from the Allevamento Cirà, Como, Italy, and starved 24–48 h before starting the experiments. The larvae were infected by injection in the last proleg of bacterial cells resuspended in 10 µl of physiological solution. Infected and mock-infected (i.e. with sterile physiological solution) larvae were incubated at 37 °C in Petri dishes and survival monitored for 42 h. Larvae were considered dead when they did not respond to gentle prodding. Statistical analyses were carried out using GraphPad Prism (GraphPad Software, San Diego, CA, USA). Survival curves were plotted using the Kaplan-Meier method, and differences in survival were calculated using the log-rank test for multiple comparisons. For measuring the bacterial titre in the hemolymph, larvae infected as described above, were sacrificed after 16 h incubation at 37 °C. The hemolymph was collected by puncturing with a needle cold-anesthetized larvae, diluted in physiological solution and plated on LD-ampicillin. Prophenoloxidase cascade activation in the hemolymph was measured as described^70^ on samples collected as described above from larvae infected with 10 µl of physiological solution containing about 2 × 10^4^ CFU or mock-infected. The larvae were sacrificed after 4 h incubation at 37 °C. Hemolymph samples obtained from 5 individuals per condition (15 µl/larva) were mixed and centrifuged at 1500 g for 10 min at 4 °C for removing hemocytes. Immediately after centrifugation, samples were diluted tenfold in TBS (50 mM Tris–HCl pH 6.8, 1 mM NaCl). 2 µl were added to 18 µl of TBS additionated with 5 mM CaCl_2_ in polystyrene 96-well microplates and incubated 20 min at room temperature. 180 µl of 2 mM dopamine in 50 mM sodium phosphate (pH 6.5) were added. Melanin formation was estimated by measuring absorbance at 490 nm over 45 min at 15-min intervals using an Ensight (PerkinElmer) microplate reader.

### Glucose quantification in growth media and *G*. *mellonella* hemolymph

Glucose concentration in growth media (LD and 2% casamino acids stock solution) and *Galleria mellonella* hemolymph was measured with the Glucose (HK) Assay Kit (Sigma-Aldrich) as detailed in Supplementary Methods.

## Electronic supplementary material

Supplementary Information

## Data Availability

Raw sequencing data are publicly available at Sequence Reads Archive under accession number PRJNA479815. Bacterial strains and other datasets presented in this study are available from the corresponding author.
