# Supplementary material for: Pseudomonas aeruginosa mutants defective in glucose uptake have pleiotropic phenotype and altered virulence in non-mammal infection models
Source: Sci Rep. 2018 Nov 15;8:16912. doi: 10.1038/s41598-018-35087-y (PMC6237876; doi:10.1038/s41598-018-35087-y)
Supplement: Supplementary file 1 — Supplementary Information [file 41598_2018_35087_MOESM1_ESM.pdf]

Matteo Raneri[1], Eva Pinatel[2], Clelia Peano[2,5], Giordano Rampioni[3], Livia Leoni[3], Irene Bianconi[4], Olivier Jousson[4], Chiara Dalmasio[1], Palma Ferrante[1] and Federica Briani[1,*]

Pseudomonas aeruginosa mutants defective in glucose uptake have pleiotropic phenotype and altered virulence in non-mammal infection models

[1]Dipartimento di Bioscienze, Università degli Studi di Milano, Italy

[2]Istituto di Tecnologie Biomediche-CNR, Segrate, Italy

[3]Dipartimento di Scienze, Università degli Studi Roma Tre, Italy

[4]Centre for Integrative Biology, Università degli Studi di Trento, Italy

This file contains (in the stated order):

Supplementary Results. Analysis of polarity of glucose uptake genes' mutations.

Supplementary Methods

Supplementary References. A comprehensive list of references cited in Supplementary Information.

Supplementary Table S1. Bacterial strains, oligonucleotides and plasmids.

Supplementary Table S2. List of differentially expressed genes with general sequencing statistics

Supplementary Table S3. Glucose responsive genes in PAO1

Supplementary Table S4. DEGs encoding putative/known transcription regulators

Supplementary Figs. S1-S6

SUPPLEMENTARY RESULTS

glt, gntP and _kguT_ deletions have not polar effect on the transcription of downstream genes

We analysed the transcription profile in the GUN mutant of *oprB, gapN* and *kguD* genes, which are located downstream of *gltK*, *gntP* and *kguT*, respectively, to verify whether the Δ*glt,* Δ*gntP* and Δ*kguT* deletions may have polar effect. We observed that the transcription pattern of *gapN* and *oprB* was very similar in the GUN mutant and in the wild type PAO1 (regardless of the presence of glucose; Supplementary Fig. S6). However, the analysis of transcription profile did not allow to draw any conclusion about the expression of *kguD*, the gene downstream of *kguT*, because the *kgu* operon was poorly transcribed in the tested strains. We thus expressed *in trans* the *kguT* gene in the Δ*kguT* mutant *via* an arabinose-inducible expression vector, and tested the ability of this strain to growth on 2-KG as sole carbon source. As shown in Supplementary Figure S6, plasmid-driven expression of *kguT* restored growth to wild type levels in the Δ*kguT* mutant in this medium. Since the *kguT* downstream gene *kguD* should be required for *P. aeruginosa* growth on 2-KG[1], this result suggests that the Δ*kguT* mutation has no polar effect on the expression of *kguD*.

SUPPLEMENTARY METHODS

Bacterial strains and plasmid construction

PAO1 deletion mutants of glucose uptake genes were constructed by gene replacement[2]. 500 bp long amplicons corresponding to the regions flanking the section to be deleted were obtained by PCR with proper oligonucleotides (i.e. *oprB*, 3287-3288 and 3289-3290; PA2291, 3291-3292 and 3304-3294; *kguT*, 3338-3339 and 3340-3341; *gntP*, 3343-3344 and 3345-3346; *gltKGF*, 3287-3367 and 3368-3369; Supplementary Table S1) and fused by overlapping PCR into a unique 1kb-long fragment, which was cloned into the BamHI-SpeI restriction sites of the suicide vector pKNG101. The recombinant plasmids (Supplementary Table S1) were constructed in *E. coli* CC118λpir and mobilized into *P. aeruginosa* by triparental conjugation[3]. Clones bearing the plasmids integrated into the chromosome were selected on streptomycin plates. The deletion mutants were selected on LD plates containing 10% sucrose as previously described[2]. Gene deletion was confirmed by PCR with proper oligonucleotides.

pGM2071 plasmid was constructed as follows. A PCR product covering the 2490689-2492060 region (i.e. the *kguT* open reading frame with the 50 bp upstream) was amplified using oligonucleotides 3401 and 3402. The product was digested with KpnI and ligated into pGM931

downstream of the *araBp* promoter, obtaining pGM2071. The plasmid was constructed in *E. coli* DH10B and transferred into PAMO108 (namely PAO1 Δ*kguT*) by transformation.

PAO1 genome annotation and RNA-Seq data analysis

To obtain a comprehensive annotation of PAO1 genome, we merged the annotations provided by the *Pseudomonas* Genome Database www.Pseudomonas.com[4] and RefSeq in the versions available on Dec 2017. The *Pseudomonas* Genome Database was selected as main reference and records present only in RefSeq annotation were manually integrated. Few genes showed the same Locus_Tag but different start and/or end among the two annotations; in such case, we choose to consider the largest gene unless if it included other annotated features. To functionally annotate the genome, we integrated KEGG and pseudoCAP information in the version provided by the *Pseudomonas* Genome Database and we merged the corresponding functional categories showing slightly different names among the two (see Supplementary Table S2).

BEDTools (v2.24.0)[5] and SAMtools (v0.1.19)[6] were adopted to verify library preparation and sequencing performances (see sequencing statistics sheet in Supplementary Table S2). A minimum of 2.8 M of reads was produced for each sample and biological replica and, on average, more than 90% of the reads resulted of high quality. Less than 1% of the mapped reads mapped on ribosomal RNAs; reads strand specificity, calculated on annotated CDS, was higher than 90% in every sample and the CDS coverage was enough for the gene expression analysis (i.e. 90% of the genes were covered by a minimum of 4 strand specific reads). To avoid double counting of reads mapping across two genes, only strand specific reads covering a CDS for at least 50% of their length were considered for gene read counts.

RT-qPCR mRNA analysis

The RNA was reverse-transcribed with the Takara PrimeScript™ RT kit (Perfect Real Time) and cDNA was used for Real-Time PCR with SYBR® Premix Ex Taq™ (Takara) and primers specific for each gene (3478-3479, *lasR*; 3480-3481, *ptxS*; 3482-3483, PA2264; 3492-3493, *fruI*; 3494-3495, *lasB*; 3511-3512, *coxB*; 3513-3514, *napA*; 3522-3523, *aruC*; 3524-3525, *hutU*; 3526-3527, PA5348; see Supplementary Table S1). 16S rRNA was used as reference gene (primers 3398-3399) to normalize Real Time PCR results and to calculate the relative fold change in gene expression with the $2^{-\Delta\Delta Ct}$ method[7].

NAD(H) quantification

Cells were harvested by centrifugation from 40 ml cultures of PAO1 and GUN strains grown as for the RNASeq. Cells were resuspended in 1 ml of cold physiological solution, split in two 1.5 ml tubes and immediately centrifuged at 16000xg for 1 min. 100 µl of either 0.2 M NAOH (for NAD^+ extraction) or 0.2 M HCl (for NADH extraction) were added to the cell pellets. Samples were incubated 10 min at 50 °C and then on ice for 10 min. 100 µl of either 0.1 M HCl (for NAD^+ extraction) or 0.1 M NaOH (for NADH extraction) were added dropwise while agitating the samples, which were then centrifuged 10 min at 16000xg. The supernatants containing NAD dinucleotide were transferred to fresh tubes. NADH and NAD^+ were immediately quantified by a cyclic assay as previously described[8]. 90-µl aliquots of a mix prepared with 1 vol of Bicine buffer (2.0 M, pH 8.0), 8 vol water, 1 vol 80 mM EDTA, 2 vol 100% ethanol, 2 vol 4.2 mM thiazolyl blue and 4 vol 16.6 mM phenazine ethosulfate were dispensed into the wells of a 96-well microtiter plate. Five microliters of either NAD^+ (Sigma) and NADH (Sigma) standard solutions or sample were added to each well and the reaction was started by the addition of 5 µl of alcohol dehydrogenase (Sigma) at 347 units/ml in 0.1 M Bicine (pH 8.0). Absorbance at 570 nm was read every 60 seconds by means an Ensight (PerkinElmer) microplate reader. Slopes of the absorbance curves over time of NADH and NAD^+ solutions were used to generate standard curves, which were used to calculate NAD concentrations (in µM) in the samples. Values were normalized for the optical density of the original cell culture sample and for NAD concentration in reference condition (i.e. PAO1 without glucose).

Secretion of pyocyanin, pyoverdine, proteases and rhamnolipids

Bacterial cultures for pyocyanin and pyoverdine extraction were grown at 37°C for 24 h in LD. Pyocyanin was extracted from the supernatant with chloroform and HCl as described[9]. The relative concentration of pyocyanin was determined as the ratio between the A_{520} of the resulting solution and the OD_{600} of the culture. Relative pyoverdine concentration[10] was determined as the ratio between the A_{405} of the culture supernatant mixed with an equal volume of 200 mM Tris-HCl (pH 8) and the OD_{600} of the culture. Extracellular proteases were tested by spotting onto casein-agar plates (1% agar supplemented with 1% casein and 150 µg/ml carbenicillin, to prevent cell growth) 2 µl of supernatants of cultures grown as described in Supplementary Fig. S4 legend and properly diluted to the same OD_{600} before cells removal by centrifugation and plating. For detection of rhamnolipids[11], overnight cultures in LD were washed in 1x PBS and resuspended in the same solution at OD_{600}= 4. 5 µl were spotted onto 1.6% M8-BactoAgar (Difco) medium supplemented with 2 mM $MgSO_4$, 0.05% (w/v) glutamic acid, 2% (v/v) glycerol, 0.5% (w/v) succinate, 0.02%

(w/v) cetyltrimethylammonium bromide (CTAB), and 0.0005% (w/v) methylene blue. Plates were incubated at 37°C for 24 h, then at room temperature for 4 days, and finally at 4°C for 24 h. The dark blue halo surrounding the cell spot due to the precipitation of CTAB and methylene blue was measured as an indication of rhamnolipid secretion.

Growth in anaerobiosis and microaerophilic conditions

A single colony was resuspended in 100 µl of 1x PBS in 96-well polystyrene microplates. Serial ten-fold dilutions were replicated on plates of LD/Agar supplemented with 100 mM KNO_3. The plates were incubated at 37°C for 16 h in aerobic conditions or for 48 h inside an anaerobic jar AnaeroJar (Oxoid) containing an AnaeroGen sachet (Oxoid). For microaerophilic growth, bacterial cultures were inoculated at OD_{600}= 0.1 in 100 µl of LD or M9-CAA in 96-well polystyrene microplates and overlaid with 50 µl of paraffin oil to prevent evaporation. The microplates were incubated static for 16 h at 37°C and the OD_{600} was measured by means of an Ensight (PerkinElmer) microplate reader.

Quantification of QS signal molecules

Levels of QS signal molecules in *P. aeruginosa* PAO1 wild type and GUN mutant culture supernatants were determined at different times during bacterial growth as previously described[12]. Briefly, the strains were grown at 37°C in shaking (200 rpm) in M9-CAA in the absence or in the presence of 0.4 % (w/v) glucose. Ten-µl of culture supernatants (or appropriate dilutions) were added to 190 µl of LB inoculated with reporter strains specific for $3OC_{12}$-HSL[13], or C_4-HSL[14] to a final OD_{600} of 0.045 in black clear-bottom 96-well microtiter plates. Microtiter plates were incubated at 37°C with gentle shaking, and the OD_{600} and relative light units (RLU) were measured after 4 h of growth for the $3OC_{12}$-HSL biosensor or 7 h of growth for the C_4-HSL biosensor. Dedicated calibration curves were generated by growing each reporter strain in the presence of increasing concentrations of the corresponding synthetic signal molecule, and these curves were used to calculate the concentration of the different QS signal molecules in each culture supernatant. Maximal QS signal molecule concentration determined during bacterial growth is reported in Fig. 4a.

Biofilm formation

Biofilm formation was assessed using the microtiter plate biofilm assay[15]. Briefly, *P. aeruginosa* PAO1 wild type an GUN mutant were grown in M9-CAA at 37°C in shaking (200 rpm) in M9-CAA in the absence or in the presence of 0.4 % (w/v) glucose for overnight (i.e. 16 h). Overnight

cultures were diluted to an OD_{600} of 0.05 in the corresponding medium, and 100 µl aliquots were transferred to a sterile 96-well polystyrene microtiter plate (6 wells per sample) and incubated at 30°C for 8 h. Planktonic cells were transferred to a sterile microtiter plate for OD_{600} measurements in a TECAN Spark 10M multilabel plate reader, while the attached cells were stained with 1% (w/v) crystal violet. After washing the wells five times with distilled water, the surface-associated dye was solubilized with 200 µl of ethanol. The A_{595} of the dye solution was measured in a TECAN Spark 10M multilabel plate reader. Adhesion units were determined as A_{595} normalized to OD_{600}.

For microscopic visualization of biofilm, *P. aeruginosa* PAO1 wild type and GUN mutant constitutively expressing GFP *via* the pMRP9-1 plasmid[16] were grown in an 8-well chamber slide, as previously described[17], with minor modifications. Briefly, bacterial cells were inoculated at an OD_{600} of 0.02 in 700 µL of M9-CAA in the absence or in the presence of 0.4 % (w/v) glucose. Cultures were incubated at 30°C for 24 h to allow the adhesion of the bacterial cells on the glass surface. To maintain bacterial viability, the medium was changed every 24 h. Biofilms formation was examined after 3 days incubation by using the Leica TCS SP5 confocal microscope. Ten random fields were examined for each sample, and representative images are reported in Fig. 4c.

Synchronization of *C. elegans*

The bleaching technique was used to synchronise *C. elegans* at the first larval stage (L1)[18], with minor modifications. Adult worms are sensitive to bleach (5% solution of sodium hypochlorite, NaClO) and die quickly, while the embryos are protected by the eggshell. *C. elegans* NGM plates were washed twice with 7 ml of M9 medium (Sigma-Aldrich). The supernatant containing the worms was transfer in a centrifuge tube and the worms pelleted for 1 min centrifugation at 1500xg. Worms' pellet was washed with until the liquid was clear. 3.5 ml of M9, 500 µl of 5 M NaOH and 1 ml of bleach were then added to the pellet. To not damage embryos, bleach-induced death was observed by using a stereomicroscope to verify the dissolution of adult worms that typically occurs within 4 minutes. Bleach was inactivated by adding M9 to a final volume of 15 ml, and the tubes centrifuged 2 minutes at 1500xg. Eggs' pellet was then washed five times with 15 ml of M9. 6 ml of M9 were then added and the tubes were placed in a dark environment at room temperature and slow agitation for two days. Synchronized worms were then gently transferred to NGM plates seeded with *E. coli* OP50 and maintained at 20° C for two days.

Glucose quantification in growth media and *G. mellonella* hemolymph

Glucose concentration in growth media (LD and 2% casamino acids stock solution) and *Galleria mellonella* hemolymph was measured with the Glucose (HK) Assay Kit (Sigma-Aldrich) following

the recommendations of the provider. Hemolymph samples obtained from 5 larvae (30 µl/larva) were mixed and centrifuged at 1500xg for 10 min at 4°C for hemocytes removal. 40 µl samples were combined with 200 µl of either Glucose Assay Reagent or 50mM Tris-HCl pH 7.5 (blank samples) and incubated 15 min at room temperature before reading the absorbance at 340 nm by means of an Ensight (PerkinElmer) microplate reader. Glucose concentration was determined by comparison with a glucose standard curve obtained by testing known dilutions of Glucose Standard Solution (Sigma-Aldrich).

SUPPLEMENTARY REFERENCES

1. del Castillo, T. *et al.* Convergent peripheral pathways catalyze initial glucose catabolism in *Pseudomonas putida*: genomic and flux analysis. *J. Bacteriol.* **189,** 5142–52 (2007).
2. Kaniga, K., Delor, I. & Cornelis, G. R. A wide-host-range suicide vector for improving reverse genetics in Gram-negative bacteria: inactivation of the *blaA* gene of *Yersinia enterocolitica*. *Gene* **109,** 137–141 (1991).
3. Goldberg, J. B. & Ohman, D. E. Cloning and expression in *Pseudomonas aeruginosa* of a gene involved in the production of alginate. *J. Bacteriol.* **158,** 1115–1121 (1984).
4. Winsor, G. L. *et al.* Enhanced annotations and features for comparing thousands of *Pseudomonas* genomes in the *Pseudomonas* genome database. *Nucleic Acids Res.* **44,** D646–D653 (2016).
5. Quinlan, A. R. & Hall, I. M. BEDTools: A flexible suite of utilities for comparing genomic features. *Bioinformatics* **26,** 841–842 (2010).
6. Li, H. *et al.* The Sequence Alignment/Map format and SAMtools. *Bioinformatics* **25,** 2078–2079 (2009).
7. Livak, K. J. & Schmittgen, T. D. Analysis of relative gene expression data using real-time quantitative PCR and the $2^{-\Delta\Delta C_T}$ Method. *Methods* **25,** 402–408 (2001).
8. Price-Whelan, A., Dietrich, L. E. P. & Newman, D. K. Pyocyanin alters redox homeostasis and carbon flux through central metabolic pathways in *Pseudomonas aeruginosa* PA14. *J. Bacteriol.* **189,** 6372–6381 (2007).
9. Essar, D. W., Eberly, L., Hadero, A. & Crawford, I. P. Identification and characterization of genes for a second anthranilate synthase in *Pseudomonas aeruginosa*: interchangeability of the two anthranilate synthases and evolutionary implications. *J. Bacteriol.* **172,** 884–900 (1990).
10. Imperi, F., Tiburzi, F. & Visca, P. Molecular basis of pyoverdine siderophore recycling in *Pseudomonas aeruginosa*. *Proc. Natl. Acad. Sci.* **106,** 20440–20445 (2009).
11. Kohler, T., Curty, L. K., Barja, F., Van Delden, C. & Pechere, J. C. Swarming of *Pseudomonas aeruginosa* is dependent on cell-to-cell signaling and requires flagella and pili. *J. Bacteriol.* **182,** 5990–5996 (2000).
12. Imperi, F. *et al.* New life for an old Drug: The anthelmintic drug niclosamide inhibits *Pseudomonas aeruginosa* quorum sensing. *Antimicrob. Agents Chemother.* **57,** 996–1005 (2013).
13. Massai, F. *et al.* A multitask biosensor for micro-volumetric detection of N-3-oxo-dodecanoyl-homoserine lactone quorum sensing signal. *Biosens. Bioelectron.* **26,** 3444–3449 (2011).
14. Duan, K. & Surette, M. G. Environmental regulation of *Pseudomonas aeruginosa* PAO1 Las and Rhl quorum-sensing systems. *J. Bacteriol.* **189,** 4827–4836 (2007).

15. Merritt, J. H., Kadouri, D. E. & O'Toole, G. A. Growing and analyzing static biofilms. *Curr. Protoc. Microbiol.* (2011). doi:10.1002/9780471729259.mc01b01s22
16. Davies, D. G. *et al.* The involvement of cell-to-cell signals in the development of a bacterial biofilm. *Science (80-.).* **280,** 295–298 (1998).
17. Jurcisek, J. A., Dickson, A. C., Bruggeman, M. E. & Bakaletz, L. O. *In vitro* Biofilm Formation in an 8-well Chamber Slide. *J. Vis. Exp.* (2011). doi:10.3791/2481
18. Porta-de-la-Riva, M., Fontrodona, L., Villanueva, A. & Cerón, J. Basic *Caenorhabditis elegans* methods: synchronization and observation. *J. Vis. Exp.* e4019 (2012). doi:10.3791/4019
19. Holloway, B. W. Genetic Recombination in *Pseudomonas aeruginosa*. *J Gen Microbiol* (1955). doi:10.1099/00221287-13-3-572
20. Herrero, M., De Lorenzo, V. & Timmis, K. N. Transposon vectors containing non-antibiotic resistance selection markers for cloning and stable chromosomal insertion of foreign genes in gram-negative bacteria. *J. Bacteriol.* **172,** 6557–6567 (1990).
21. Grant, S. G. N., Jessee, J., Bloom, F. R. & Hanahan, D. Differential plasmid rescue from transgenic mouse DNAs into *Escherichia coli* methylation-restriction mutants. *Proc. Natl. Acad. Sci. U. S. A.* **87,** 4645–4649 (1990).
22. Boyer, H. W. & Roulland-dussoix, D. A complementation analysis of the restriction and modification of DNA in *Escherichia coli*. *J. Mol. Biol.* **41,** 459–472 (1969).
23. Figurski, D. H., Meyer, R. J. & Helinski, D. R. Suppression of colE1 replication properties by the Inc P-1 plasmid RK2 in hybrid plasmids constructed *in vitro*. *J. Mol. Biol.* **133,** 295–318 (1979).
24. Delvillani, F. *et al.* Tet-Trap, a genetic approach to the identification of bacterial RNA thermometers: application to *Pseudomonas aeruginosa*. *RNA* **20,** 1963–1976 (2014).
25. Lu, C. D., Yang, Z. & Li, W. Transcriptome analysis of the ArgR regulon in *Pseudomonas aeruginosa*. *J. Bacteriol.* **186,** 3855–3861 (2004).
26. Udaondo, Z., Ramos, J.-L., Segura, A., Krell, T. & Daddaoua, A. Regulation of carbohydrate degradation pathways in *Pseudomonas* involves a versatile set of transcriptional regulators. *Microb. Biotechnol.* **11,** 442–454 (2018).
27. Chavarría, M. *et al.* Fructose 1-phosphate is the one and only physiological effector of the Cra (FruR) regulator of *Pseudomonas putida*. *FEBS Open Bio* **4,** 377–386 (2014).
28. Ochsner, U. A., Johnson, Z. & Vasil, M. L. Genetics and regulation of two distinct haem-uptake systems, *phu* and *has*, in *Pseudomonas aeruginosa*. *Microbiology* **146,** 185–198 (2000).

Supplementary Table S1. Bacterial strains, oligonucleotides and plasmids

Bacteria

**Strain**	**Mutation**	**Deletion**[a]	**Reference**
P. aeruginosa			
PAO1	na	none	19
PAMO104	Δ*oprB*	Δ1 (3575912-3577276)	this work
PAMO105	ΔPA2291	Δ2 (2521224-2522643)	this work
PAMO106	Δ*oprB* ΔPA2291	Δ1 and Δ2	this work
PAMO107	Δ*gntP*	Δ3 (2560762-2562114)	this work
PAMO108	Δ*kguT*	Δ4 (2490738-2492045)	this work
PAMO109	Δ*gntP* Δ*kguT*	Δ3 and Δ4	this work
PAMO110	Δ*gltKGF* Δ*gntP* Δ*kguT*	Δ3, Δ4 and Δ5	this work
PAMO111	Δ*gltKGF*	Δ5 (3577778-3580283)	this work
E. coli			
CC118λpir			20
DH10B			21
HB101			22

Oligonucleotides

**Name**	**5'→3' Sequence**[b]	**Coordinates**[a]
3287	GGGGGATCCTCATCGGTTCGCCGCCGA	3577777 - 3577760
3288	TTCCAGCGTCCTCGTGGTTG	3577277 - 3577296
3289	CACGAGGACGCTGGAATCGTCGCGTTGCCTGCTC	3577292 - 3577277
		3575911 - 3575894
3290	GGGACTAGTGCGGCCATTCGCTGCCG	3575412 - 3575428
3291	GGGGGATCCGCAATGCCGGGCGCAGC	2523117 - 2523101

3292	GCGAACGCTTCCTCGTTGC	2522617 - 2522635
3294	GGGACTAGTGTCGGCCAGCAGCGGGC	2520757 - 2520773
3304	GATCCAGACGGTGTTCTAGGC	2521276 - 2521256
3316	CGGGGTCGACGAGGTCGACAACGCG	2521318 - 2521294
3317	CTGGTGGCGGGGATCAAGATCCAGACGGTG	2521293 - 2521264
3338	GGGGGATCCCATGATCGCCGAGATCAACGC	2490261 - 2490281
3339	GGTCGGGTATCTCCTGAGC	2490737 - 2490719
3340	GCTCAGGAGATACCCGACCCCGACTCCGGAGCATCCG	2490719 - 2490737
		2492046 - 2492063
3341	GGGACTAGTCCCATGCCGACGATACCGAG	2492521 -2492502
3342	CAACCCGCACGCCGACAAGCGCG	2491967 - 2491989
3343	GGGGGATCCACATCCGCAAGATGAGCGCC	2560274 - 2560293
3344	GGAGGGCTCTCCTTTTGTCG	2560761 - 2560742
3345	CGACAAAAGGAGAGCCCTCCGCACCAGCCCGACCGGA	2560742 - 2560761
		2562115 - 2562131
3346	GGGACTAGTCGAGGAATACCGGGCTGCGT	2562609 - 2562590
3347	GACCGCGATGGAGACCATCCTCTCCG	2562045 - 2562070
3354	TCGCTGTGGCCCAGCGAGCGCCG	2490238 - 2490260
3355	CGGGCCAGGGCCTCGCCGATGC	2492524 - 2492545
3367	GGGACTAGTTGGTCTAGGCAGTACGAAAGGAT	3577297 - 3577319
3368	TCGGCGGCGAACCGATGAGCGTTTTCTCGCGTGCGAAG	3577760 - 3577777
		3580284 - 3580303
3369	GGGGTCGACAGCAACGCGGAGAACCGCAA	3580722 - 3580703
3370	ACTCGCTGGTGATGTTCAAGCT	3580745 - 3580724
3371	TATTCCGAACTGCGAGGCAAGC	3579383 - 3579362
3372	TTGTGCAGGTTGCGCAGTTCG	3578449 - 3578469
3401	GGGGGTACCATGCTCCGGAGTCGGTCAG	2492060 - 2492042
3402	GGGGGTACCGCGGCACCTGTTGCGACAA	2490689 - 2490707

3478	AGCACGAGTTCTTCGAGGAAG	1558463 - 1558483
3479	GTTTTCCGCTTCCACGCTGA	1558578 - 1558559
3480	AACACGGCTACAGCCTGGTG	2488148 - 2488167
3481	ACGATCAGTCCTTCGACGTTGTA	2488250 - 2488228
3482	CATCGACCGACAGATGGAAAC	2493478 - 2493498
3483	GTGGCACCAGCTTCAACTGAT	2493590 - 2493570
3492	ACCGAACAGTTGCAGCAGGC	3992539 - 3992520
3493	AGGTTGGCGCAGACTTCCAC	3992412 - 3992431
3494	AAGACCGGCGAAGTGCTCGA	4169934 - 4169915
3495	TAGTCGCTACCGTAGGTGTACTT	4169831 - 4169853
3511	GAGAGCACCACGGTGGAAAT	127618 - 127637
3512	GGAAGTGTCGTAGATGTGGATC	127722 - 127701
3513	ACATCAAGGCCGAGGTCAAC	1275599 - 1275580
3514	AACTTGCCGTCCTTCATGCG	1275482 - 1275501
3522	AACTGATCGATTTCGCCGGC	978037 - 978056
3523	GACGTTGGAAACGTGCCAGAT	978149 - 978129
3524	GCATGCTGATGAACAACCTCG	5744659 - 5744639
3525	TGTCGTAGCACTCCCAGTTG	5744555 - 5744574
3526	AAGGCCGATCTCACCAAGGAA	6017386 - 6017366
3527	GTTGCAGGAAGGTGCCGAAA	6017266 - 6017285

Plasmids

**Name**	**Relevant characteristics**[c]	**Reference**
pRK2013	ColE1 *ori* tra^+ mob^+ Km^r	23
pKNG101	Suicide vector in *P. aeruginosa sacB* Sm^r	2
pGM931	pHERD20T derivative carrying *araBp*-tΩ region	24
pGM2050	pKNG101 derivative, carries *oprB* US (3577277-3577777) and DS (3575412-3575911) fragments	this work

pGM2051	pKNG101 derivative, carries PA2291 US (2522617- - 2523117) and DS (2520757-2521257) fragments	this work
pGM2059	pKNG101 derivative, carries *gntP* US (2560274-2560761) and DS (2562115-2562609) fragments	this work
pGM2060	pKNG101 derivative, carries *kguT* US (2490261-2490737) and DS (2492046-2492545) fragments	this work
pGM2066	pKNG101 derivative, carries *gltF* US (3580284-3580722) and *gltK* (3577297-3577777) fragments	this work
pGM2071	pGM931 derivative, carries *kguT* (2490689-2492060) under *araBp* control	this work
pMRP9-1		16

[a]Coordinates refer to *P. aeruginosa* PAO1 NCBI Reference Sequence: NC_002516.2.
[b]Underlined characters, restriction sites.
[c]US, upstream; DS, downstream

Supplementary Table S2. List of differentially expressed genes and general statistics

Locus[2]	Log$_2$ Fold Change[1]			Name	Description
	GUN+ *vs.* PAO1+	GUN+ *vs.* PAO1	PAO1+ *vs.* PAO1		
PA0009	-0,94	**-1,05**	-0,11	glyQ	glycyl-tRNA synthetase alpha chain
PA0039	**1,17**	**1,39**	0,22	NA	hypothetical protein
PA0045	**-1,62**	**-1,93**	-0,31	NA	hypothetical protein
PA0046	**-1,97**	**-2,00**	-0,04	NA	hypothetical protein
PA0047	-1,20	**-1,29**	-0,09	NA	hypothetical protein
PA0049	**-3,54**	**-4,25**	-0,71	NA	hypothetical protein
PA0052	**1,67**	0,95	-0,72	NA	hypothetical protein
PA0059	**1,37**	**1,93**	0,56	osmC	osmotically inducible protein OsmC
PA0082	-0,73	**-1,38**	-0,65	tssA1	TssA1
PA0083	-0,75	**-1,28**	-0,53	tssB1	TssB1
PA0084	-0,79	**-1,22**	-0,42	tssC1	TssC1
PA0090	-0,76	**-1,19**	-0,43	clpV1	ClpV1
PA0105	**3,03**	**2,69**	-0,33	coxB	cytochrome c oxidase, subunit II
PA0106	**3,06**	**2,94**	-0,12	coxA	cytochrome c oxidase, subunit I
PA0107	**2,40**	**2,60**	0,19	NA	conserved hypothetical protein
PA0108	**2,38**	**2,19**	-0,19	coIII	cytochrome c oxidase, subunit III
PA0110	**2,85**	**2,11**	-0,75	NA	hypothetical protein
PA0111	**2,44**	**1,97**	-0,47	NA	hypothetical protein
PA0112	**1,91**	**1,48**	-0,43	NA	hypothetical protein
PA0113	**2,14**	**1,36**	-0,78	NA	probable cytochrome c oxidase assembly factor
PA0122	**2,61**	**1,91**	-0,70	rahU	rahU
PA0128	**-1,21**	**-1,27**	-0,06	phnA	conserved hypothetical protein
PA0141	**-2,05**	**-1,50**	0,55	NA	conserved hypothetical protein
PA0176	**1,64**	**1,55**	-0,09	aer2	aerotaxis transducer Aer2
PA0177	**1,57**	**1,50**	-0,07	NA	probable purine-binding chemotaxis protein
PA0178	**1,81**	**1,26**	-0,55	NA	probable two-component sensor
PA0179	**1,44**	**1,10**	-0,34	NA	probable two-component response regulator
PA0180	**1,30**	1,02	-0,27	cttP	chemotactic transducer for trichloroethylene [positive chemotaxis], CttP
PA0208	**3,19**	**2,22**	-0,97	mdcA	malonate decarboxylase alpha subunit
PA0209	**2,63**	**2,39**	-0,24	mdcB	conserved hypothetical protein
PA0210	**1,67**	1,63	-0,04	mdcC	malonate decarboxylase delta subunit
PA0211	**1,99**	**1,88**	-0,11	mdcD	malonate decarboxylase beta subunit
PA0212	**2,05**	**1,74**	-0,31	mdcE	malonate decarboxylase gamma subunit
PA0213	**1,95**	**1,50**	-0,44	mdcG	hypothetical protein

PA0214	**2,55**	**2,03**	-0,52	mdcH	probable acyl transferase
PA0215	**1,60**	**1,59**	0,00	madL	malonate transporter MadL
PA0216	**1,58**	1,07	-0,51	madM	malonate transporter MadM
PA0256	**1,03**	0,80	-0,23	NA	hypothetical protein
PA0277	**-1,37**	**-1,52**	-0,15	NA	conserved hypothetical protein
PA0285	-0,76	**-1,07**	-0,31	NA	conserved hypothetical protein
PA0316	**-1,08**	-0,84	0,24	serA	D-3-phosphoglycerate dehydrogenase
PA0329	**1,17**	**1,67**	0,50	NA	conserved hypothetical protein
PA0355	**2,02**	**2,43**	0,41	pfpI	protease PfpI
PA0365	**1,17**	0,64	-0,53	NA	hypothetical protein
PA0394	-0,83	**-1,19**	-0,37	yggS	conserved hypothetical protein
PA0447	**-1,21**	-0,73	0,48	gcdH	glutaryl-CoA dehydrogenase
PA0459	0,93	**1,16**	0,22	clpC	probable ClpA/B protease ATP binding subunit
PA0484	**1,62**	**1,26**	-0,36	NA	conserved hypothetical protein
PA0510	**-2,01**	**-1,75**	0,25	nirE	NirE
PA0511	**-2,14**	**-1,77**	0,37	nirJ	heme d1 biosynthesis protein NirJ
PA0512	**-1,80**	**-1,58**	0,22	nirH	NirH
PA0513	**-1,33**	**-1,39**	-0,06	nirG	NirG
PA0517	**-1,35**	-0,91	0,44	nirC	probable c-type cytochrome precursor
PA0519	**-1,58**	**-1,18**	0,40	nirS	nitrite reductase precursor
PA0527	**-1,46**	-0,64	0,82	dnr	transcriptional regulator Dnr
PA0546	**1,09**	**1,16**	0,08	metK	methionine adenosyltransferase
PA0547	**1,23**	**1,04**	-0,19	NA	probable transcriptional regulator
PA0551	-0,52	**-1,19**	-0,66	epd	D-erythrose 4-phosphate dehydrogenase
PA0563	-0,68	**-1,05**	-0,37	NA	conserved hypothetical protein
PA0575	**1,34**	1,10	-0,24	NA	conserved hypothetical protein
PA0576	**-1,21**	**-1,12**	0,09	rpoD	sigma factor RpoD
PA0577	**-1,09**	**-1,23**	-0,13	dnaG	DNA primase
PA0579	-1,04	**-1,15**	-0,11	rpsU	30S ribosomal protein S21
PA0586	**2,09**	**1,87**	-0,22	ycgB	conserved hypothetical protein
PA0587	**2,29**	**1,90**	-0,39	yeaH	conserved hypothetical protein
PA0588	**2,27**	**2,02**	-0,26	yeaG	conserved hypothetical protein
PA0654	**-2,26**	**-2,24**	0,02	speD	S-adenosylmethionine decarboxylase proenzyme
PA0663	-0,72	**-1,05**	-0,33	NA	hypothetical protein
PA0708	**1,90**	**1,65**	-0,25	NA	probable transcriptional regulator
PA0709	**-1,07**	**-1,11**	-0,04	NA	hypothetical protein
PA0710	**-1,75**	**-1,94**	-0,19	gloA2	lactoylglutathione lyase
PA0713	**-1,93**	**-1,97**	-0,04	NA	hypothetical protein
PA0714	**-1,70**	**-1,36**	0,34	NA	hypothetical protein
PA0730	-0,87	**-1,05**	-0,17	NA	probable transferase
PA0745	**1,02**	0,80	-0,22	NA	probable enoyl-CoA hydratase/isomerase
PA0779	**-1,50**	**-1,45**	0,05	asrA	AsrA

PA0783	**-1,08**	-0,85	0,24	putP	sodium/proline symporter PutP
PA0789	**-1,59**	**-2,08**	-0,48	NA	probable amino acid permease
PA0792	**1,40**	**1,55**	0,16	prpD	propionate catabolic protein PrpD
PA0798	**1,82**	**1,22**	-0,61	pmtA	phospholipid methyltransferase
PA0852	**1,42**	**1,02**	-0,39	cbpD	chitin-binding protein CbpD precursor
PA0852.1	**1,37**	1,00	-0,37	NA	Uncharacterized protein
PA0866	**1,85**	**1,16**	-0,69	aroP2	aromatic amino acid transport protein AroP2
PA0888	**-1,49**	**-2,80**	**-1,31**	aotJ	arginine/ornithine binding protein AotJ
PA0889	-1,09	**-2,04**	-0,94	aotQ	arginine/ornithine transport protein AotQ
PA0890	-1,25	**-1,80**	-0,55	aotM	arginine/ornithine transport protein AotM
PA0891	**-1,54**	**-2,61**	-1,06	aotO	hypothetical protein
PA0892	**-1,31**	**-1,68**	-0,37	aotP	arginine/ornithine transport protein AotP
PA0893	-0,69	**-1,39**	-0,69	argR	transcriptional regulator ArgR
PA0895	**-1,56**	**-2,63**	-1,06	aruC	N2-Succinylornithine 5-aminotransferase (SOAT) = N2-acetylornithine 5-aminotransferase (ACOAT)
PA0896	-1,04	**-2,23**	-1,19	aruF	subunit I of arginine N2-succinyltransferase = ornithine N2-succinyltransferase
PA0897	**-1,69**	**-2,91**	-1,22	aruG	subunit II of arginine N2-succinyltransferase = ornithine N2-succinyltransferase
PA0898	**-1,45**	**-2,34**	-0,89	aruD	N-Succinylglutamate 5-semialdehyde dehydrogenase
PA0899	-1,13	**-1,82**	-0,69	aruB	N2-Succinylarginine dihydrolase
PA0901	-0,89	**-1,15**	-0,26	aruE	N-Succinylglutamate desuccinylase
PA0916	-0,93	**-1,50**	-0,56	yliG	conserved hypothetical protein
PA0945	**-1,08**	**-1,09**	-0,01	purM	phosphoribosylaminoimidazole synthetase
PA0956	-0,89	**-1,11**	-0,22	proS	prolyl-tRNA synthetase
PA0969	**-1,13**	-0,80	0,33	tolQ	TolQ protein
PA1011	-0,91	**-1,12**	-0,21	NA	hypothetical protein
PA1041	**2,94**	**2,39**	-0,55	NA	probable outer membrane protein precursor
PA1070	-0,58	**-1,15**	-0,57	braG	branched-chain amino acid transport protein BraG
PA1071	-0,56	**-1,24**	-0,68	braF	branched-chain amino acid transport protein BraF
PA1123	-1,22	**-1,33**	-0,11	NA	hypothetical protein
PA1130	**2,15**	**1,40**	-0,75	rhlC	rhamnosyltransferase 2
PA1135	1,02	**1,29**	0,27	yedU	conserved hypothetical protein
PA1155	**-1,13**	-0,88	0,24	nrdB	NrdB, tyrosyl radical-harboring component of class Ia ribonucleotide reductase
PA1156	**-1,05**	-0,99	0,06	nrdA	NrdA, catalytic component of class Ia ribonucleotide reductase

PA1166	**1,58**	**1,59**	0,02	NA	hypothetical protein
PA1168	**1,97**	**1,88**	-0,10	NA	hypothetical protein
PA1169	**1,73**	**1,51**	-0,22	NA	probable lipoxygenase
PA1172	**1,79**	**2,06**	0,27	napC	cytochrome c-type protein NapC
PA1173	**1,59**	**1,63**	0,04	napB	cytochrome c-type protein NapB precursor
PA1174	**2,26**	**1,86**	-0,40	napA	periplasmic nitrate reductase protein NapA
PA1175	**2,12**	**1,74**	-0,39	napD	NapD protein of periplasmic nitrate reductase
PA1176	**2,31**	**1,89**	-0,43	napF	ferredoxin protein NapF
PA1177	**1,50**	**1,70**	0,20	napE	periplasmic nitrate reductase protein NapE
PA1190	**2,08**	**2,11**	0,02	yohC	conserved hypothetical protein
PA1196	**-1,46**	-0,61	0,86	ddaR	transcriptional regulator DdaR
PA1211	1,63	**1,82**	0,19	NA	hypothetical protein
PA1212	**2,29**	**2,50**	0,20	NA	probable major facilitator superfamily (MFS) transporter
PA1213	**3,77**	**2,90**	-0,87	NA	hypothetical protein
PA1214	**3,31**	**2,88**	-0,42	NA	hypothetical protein
PA1215	**3,15**	**1,94**	-1,20	NA	hypothetical protein
PA1216	**3,56**	**2,91**	-0,66	NA	hypothetical protein
PA1217	**3,21**	**2,62**	-0,58	NA	probable 2-isopropylmalate synthase
PA1218	**3,13**	**2,36**	-0,78	NA	hypothetical protein
PA1219	**1,96**	**1,75**	-0,20	NA	hypothetical protein
PA1220	**3,09**	**2,68**	-0,41	NA	hypothetical protein
PA1221	**2,98**	**2,46**	-0,52	NA	hypothetical protein
PA1228	**-1,81**	**-1,59**	0,22	NA	hypothetical protein
PA1245	**1,25**	**1,28**	0,04	aprX	AprX
PA1246	**1,69**	**1,51**	-0,17	aprD	alkaline protease secretion protein AprD
PA1247	**1,35**	**1,16**	-0,19	aprE	alkaline protease secretion protein AprE
PA1248	**1,22**	0,82	-0,41	aprF	Alkaline protease secretion outer membrane protein AprF precursor
PA1249	**2,41**	**1,80**	-0,61	aprA	alkaline metalloproteinase precursor
PA1250	**1,23**	**1,11**	-0,12	aprI	alkaline proteinase inhibitor AprI
PA1256	**1,62**	0,97	-0,65	lhpO	ABC transporter ATP-binding protein, LhpO
PA1317	-1,00	**-1,19**	-0,19	cyoA	cytochrome o ubiquinol oxidase subunit II
PA1318	-1,01	**-1,12**	-0,11	cyoB	cytochrome o ubiquinol oxidase subunit I
PA1323	**2,13**	**2,63**	0,50	NA	hypothetical protein
PA1324	**2,22**	**2,55**	0,33	NA	hypothetical protein
PA1353	**2,07**	**2,31**	0,24	NA	hypothetical protein
PA1361	-0,73	**-1,26**	-0,54	norM	NorM
PA1404	**2,00**	**1,80**	-0,19	NA	hypothetical protein
PA1471	0,95	**1,33**	0,38	NA	hypothetical protein

PA1546	**-1,16**	-0,33	0,82	hemN	oxygen-independent coproporphyrinogen III oxidase
PA1549	-0,81	**-1,21**	-0,40	fixI	probable cation-transporting P-type ATPase
PA1551	-0,93	**-1,23**	-0,30	fixG	probable ferredoxin
PA1552	-0,82	**-1,04**	-0,22	ccoP1	Cytochrome c oxidase, cbb3-type, CcoP subunit
PA1554	-0,86	**-1,19**	-0,33	ccoN1	Cytochrome c oxidase, cbb3-type, CcoN subunit
PA1555	**-2,37**	**-1,93**	0,43	ccoP2	Cytochrome c oxidase, cbb3-type, CcoP subunit
PA1555.1	**-2,24**	**-2,21**	0,03	ccoQ2	Cytochrome c oxidase, cbb3-type, CcoQ subunit
PA1556	**-2,10**	**-1,44**	0,66	ccoO2	Cytochrome c oxidase, cbb3-type, CcoO subunit
PA1557	**-2,03**	**-1,35**	0,68	ccoN2	Cytochrome c oxidase, cbb3-type, CcoN subunit
PA1562	0,98	**1,02**	0,04	acnA	aconitate hydratase 1
PA1582	-0,65	**-1,07**	-0,43	sdhD	succinate dehydrogenase (D subunit)
PA1583	-0,86	**-1,27**	-0,41	sdhA	succinate dehydrogenase (A subunit)
PA1584	-0,81	**-1,20**	-0,38	sdhB	succinate dehydrogenase (B subunit)
PA1592	**1,12**	**1,71**	0,59	NA	hypothetical protein
PA1596	**-1,80**	**-1,62**	0,18	htpG	heat shock protein HtpG
PA1617	**1,13**	**1,10**	-0,03	NA	probable AMP-binding enzyme
PA1643a	**1,50**	1,15	-0,35	NA	NA
PA1673	**-1,73**	**-1,29**	0,43	NA	hypothetical protein
PA1687	**-1,13**	-0,80	0,33	speE	spermidine synthase
PA1732	**1,40**	0,82	-0,58	NA	conserved hypothetical protein
PA1745	**1,70**	1,27	-0,44	NA	hypothetical protein
PA1750	-0,96	**-1,14**	-0,17	NA	phospho-2-dehydro-3-deoxyheptonate aldolase
PA1761	**1,62**	**1,21**	-0,40	NA	hypothetical protein
PA1784	**2,16**	**1,61**	-0,55	NA	hypothetical protein
PA1791	**-1,22**	**-1,49**	-0,27	NA	hypothetical protein
PA1805	-0,96	**-1,11**	-0,15	ppiD	peptidyl-prolyl cis-trans isomerase D
PA1818	-0,66	**-1,86**	-1,20	ldcA,cadA	lysine decarboxylase
PA1830	0,59	**1,20**	0,61	NA	hypothetical protein
PA1837a	**1,58**	1,06	-0,51	NA	NA
PA1838	**-1,07**	-0,42	0,64	cysI	sulfite reductase
PA1839	-0,88	**-1,34**	-0,46	NA	hypothetical protein
PA1869	**1,46**	0,77	-0,69	NA	probable acyl carrier protein
PA1871	**2,45**	**1,75**	-0,70	lasA	LasA protease precursor
PA1874	**1,46**	1,03	-0,43	NA	hypothetical protein
PA1880	**1,56**	**1,21**	-0,35	NA	probable oxidoreductase
PA1881	**1,30**	**1,07**	-0,23	NA	probable oxidoreductase
PA1887	**1,79**	**2,26**	0,47	NA	hypothetical protein
PA1888	**2,46**	**2,62**	0,16	NA	hypothetical protein
PA1894	**1,15**	0,71	-0,44	NA	hypothetical protein

PA1895	**1,12**	0,55	-0,57	NA	hypothetical protein
PA1897	**1,42**	0,93	-0,49	NA	hypothetical protein
PA1899	**4,48**	**3,32**	-1,16	phzA2	probable phenazine biosynthesis protein
PA1900	**4,30**	**3,01**	-1,28	phzB2	probable phenazine biosynthesis protein
PA1927	**1,80**	**1,38**	-0,42	metE	5-methyltetrahydropteroyltriglutamate-homocysteine S-methyltransferase
PA1930	**1,82**	**2,10**	0,28	NA	probable chemotaxis transducer
PA1946	**1,17**	**1,08**	-0,09	rbsB	binding protein component precursor of ABC ribose transporter
PA1951	**2,00**	**1,56**	-0,44	fapF	FapF
PA1964	**-1,20**	-1,06	0,14	ybiT	probable ATP-binding component of ABC transporter
PA2018	**1,50**	**1,41**	-0,08	mexY	Resistance-Nodulation-Cell Division (RND) multidrug efflux transporter MexY
PA2042	-0,78	**-1,71**	-0,93	ygjU	probable transporter (membrane subunit)
PA2066	**2,32**	**1,99**	-0,33	NA	hypothetical protein
PA2068	**2,61**	**2,33**	-0,28	NA	probable major facilitator superfamily (MFS) transporter
PA2069	**3,92**	**2,50**	-1,42	NA	probable carbamoyl transferase
PA2071	**1,29**	**1,22**	-0,07	fusA2	elongation factor G
PA2072	**2,00**	**1,72**	-0,27	NA	conserved hypothetical protein
PA2109	**-2,42**	**-2,46**	-0,04	NA	hypothetical protein
PA2111	**-3,35**	**-3,42**	-0,08	NA	hypothetical protein
PA2112	**-1,93**	**-2,07**	-0,14	NA	conserved hypothetical protein
PA2113	-1,57	**-2,23**	-0,66	opdO	pyroglutamate porin OpdO
PA2142a	1,62	**2,05**	0,43	NA	NA
PA2164	**1,58**	1,29	-0,29	NA	probable glycosyl hydrolase
PA2167	**1,47**	**1,51**	0,04	NA	hypothetical protein
PA2171	**1,52**	**2,18**	0,66	NA	hypothetical protein
PA2177	1,07	**1,42**	0,35	NA	probable sensor/response regulator hybrid
**PA2262**	**-1,86**	-1,42	0,43	kguT	probable 2-ketogluconate transporter
PA2264	-1,13	0,62	**1,75**	NA	conserved hypothetical protein
PA2265	-0,84	0,93	**1,77**	gad	gluconate dehydrogenase
PA2290	-0,48	**1,20**	**1,68**	gcd	glucose dehydrogenase
PA2291	-0,09	**1,79**	**1,88**	oprB2; opbA	probable glucose-sensitive porin
PA2300	**1,96**	**1,28**	-0,68	chiC	chitinase
PA2302	**1,41**	**1,28**	-0,13	ambE	AmbE
PA2303	**1,17**	0,96	-0,20	ambD	AmbD
PA2304	**1,18**	**1,22**	0,04	ambC	AmbC
PA2320	**-1,67**	0,20	**1,88**	gntR	transcriptional regulator GntR
PA2321	**-5,62**	-0,34	**5,28**	gntV; gntK; gnuK	gluconokinase
**PA2322**	**-5,87**	-0,72	**5,15**	gntP	gluconate permease

PA2323	**-1,62**	**3,09**	**4,71**	gapN; gapB	probable glyceraldehyde-3-phosphate dehydrogenase
PA2329	1,13	**1,29**	0,15	NA	probable ATP-binding component of ABC transporter
PA2331	**1,67**	**1,47**	-0,20	NA	hypothetical protein
PA2363	**1,44**	1,14	-0,30	hsiJ3	HsiJ3
PA2365	**2,55**	**2,00**	-0,55	hsiB3	HsiB3
PA2366	**2,79**	**2,10**	-0,69	hsiC3	HsiC3
PA2367	**2,66**	**2,11**	-0,55	hcp3	Hcp3
PA2369	**2,48**	**2,03**	-0,45	hsiG3	HsiG3
PA2371	**2,04**	**1,32**	-0,73	clpV3	ClpV3
PA2372	**2,25**	**1,74**	-0,52	NA	hypothetical protein
PA2373	**1,58**	**1,10**	-0,48	vgrG3	VgrG3
PA2375	1,29	**1,62**	0,33	NA	hypothetical protein
PA2385	**1,33**	**1,83**	0,50	pvdQ	3-oxo-C12-homoserine lactone acylase PvdQ
PA2386	**1,49**	**2,40**	0,91	pvdA	L-ornithine N5-oxygenase
PA2392	**1,19**	**1,57**	0,38	pvdP	PvdP
PA2393	**1,50**	**2,10**	0,59	NA	putative dipeptidase
PA2394	**1,22**	**2,00**	0,78	pvdN	PvdN
PA2396	**1,37**	**2,09**	0,72	pvdF	pyoverdine synthetase F
PA2397	**1,21**	**1,43**	0,22	pvdE	pyoverdine biosynthesis protein PvdE
PA2399	0,87	**1,31**	0,44	pvdD	pyoverdine synthetase D
PA2400	0,60	**1,02**	0,42	pvdJ	PvdJ
PA2402	**1,02**	**1,50**	0,48	NA	probable non-ribosomal peptide synthetase
PA2412	1,42	**2,21**	0,79	NA	conserved hypothetical protein
PA2413	**1,42**	**2,09**	0,68	pvdH	L-2,4-diaminobutyrate:2-ketoglutarate 4-aminotransferase, PvdH
PA2414	1,25	**1,53**	0,28	NA	L-sorbosone dehydrogenase
PA2424	**1,15**	**1,86**	0,71	pvdL	PvdL
PA2426	0,96	**2,27**	1,31	pvdS	sigma factor PvdS
PA2433	**2,07**	**2,59**	0,52	NA	hypothetical protein
PA2441	1,03	**1,66**	0,63	NA	hypothetical protein
PA2486	1,00	**1,23**	0,23	NA	hypothetical protein
PA2504	**1,49**	**1,83**	0,35	NA	hypothetical protein
PA2512	-0,75	**-1,46**	-0,71	antA	anthranilate dioxygenase large subunit
PA2513	-0,77	**-1,41**	-0,64	antB	anthranilate dioxygenase small subunit
PA2514	-1,05	**-1,73**	-0,67	antC	anthranilate dioxygenase reductase
PA2544	1,19	**1,39**	0,20	NA	hypothetical protein
PA2562	**1,27**	**1,49**	0,21	NA	hypothetical protein
PA2564	**1,60**	1,09	-0,50	tam	hypothetical protein
PA2566	**2,26**	**1,39**	-0,87	NA	conserved hypothetical protein
PA2570	**2,23**	**1,61**	-0,61	lecA	LecA
PA2571	**1,24**	1,00	-0,23	NA	probable two-component sensor
PA2572	**1,51**	**1,46**	-0,05	NA	probable two-component response

					regulator
PA2573	**2,36**	**1,87**	-0,49	NA	probable chemotaxis transducer
PA2579	-0,55	**-1,06**	-0,52	kynA	L-Tryptophan:oxygen 2,3-oxidoreductase (decyclizing) KynA
PA2584	-0,59	**-1,12**	-0,53	pgsA	CDP-diacylglycerol--glycerol-3-phosphate 3-phosphatidyltransferase
PA2622	**1,55**	**1,64**	0,09	cspD	cold-shock protein CspD
PA2624	**-1,49**	**-1,21**	0,29	idh	isocitrate dehydrogenase
PA2629	**-1,25**	**-1,35**	-0,10	purB	adenylosuccinate lyase
PA2630	**-1,49**	-1,03	0,46	ycfD	conserved hypothetical protein
PA2634	**1,01**	0,58	-0,43	aceA	isocitrate lyase AceA
PA2639	**-1,02**	**-1,04**	-0,01	nuoD	NADH dehydrogenase I chain C,D
PA2641	**-1,17**	**-1,26**	-0,09	nuoF	NADH dehydrogenase I chain F
PA2647	**-1,02**	-0,99	0,03	nuoL	NADH dehydrogenase I chain L
PA2722	**1,36**	1,22	-0,14	NA	hypothetical protein
PA2740	**-1,12**	-0,90	0,22	pheS	phenylalanyl-tRNA synthetase, alpha-subunit
PA2743	-0,87	**-1,19**	-0,32	infC	translation initiation factor IF-3
PA2747	**1,99**	**1,88**	-0,11	NA	hypothetical protein
PA2754	0,54	**1,70**	1,17	NA	conserved hypothetical protein
PA2760	-0,72	**-1,08**	-0,35	oprQ	OprQ
PA2765	-0,72	**-1,01**	-0,29	NA	hypothetical protein
PA2771	**1,97**	**1,62**	-0,35	NA	diguanylate cyclase with a self-blocked I-site, Dcsbis
PA2787	1,11	**1,73**	0,61	cpg2	carboxypeptidase G2 precursor
PA2815	**1,44**	**1,34**	-0,10	yafH	probable acyl-CoA dehydrogenase
PA2851	**-1,28**	**-1,20**	0,07	efp	translation elongation factor P
PA2853	0,45	**1,19**	0,74	oprI	Outer membrane lipoprotein OprI precursor
PA2862	**1,86**	**1,85**	-0,02	lipA	lactonizing lipase precursor
PA2863	**1,48**	**1,36**	-0,12	lipH	lipase modulator protein
PA2864	**1,53**	**1,48**	-0,05	NA	conserved hypothetical protein
PA2911	**-1,03**	**-1,02**	0,00	NA	probable TonB-dependent receptor
PA2937	**2,48**	**2,03**	-0,45	NA	hypothetical protein
PA2939	**3,88**	**3,23**	-0,66	pepB	probable aminopeptidase
PA2950	**-1,00**	**-1,03**	-0,03	pfm¦	proton motive force protein, PMF
PA2967	**-1,11**	**-1,33**	-0,22	fabG	3-oxoacyl-[acyl-carrier-protein] reductase
PA2968	**-1,01**	-0,97	0,04	fabD	malonyl-CoA-[acyl-carrier-protein] transacylase
PA2969	**-1,52**	**-1,68**	-0,16	plsX	fatty acid biosynthesis protein PlsX
PA2971	-0,82	**-1,30**	-0,48	yceD	conserved hypothetical protein
PA2976	**-1,05**	**-1,09**	-0,04	rne	ribonuclease E
PA2995	-0,80	**-1,09**	-0,30	nqrE	Na+-translocating NADH:quinone oxidoreductase subunit Nqr5
PA2997	**-1,04**	**-1,14**	-0,10	nqrC	Na+-translocating NADH:ubiquinone oxidoreductase subunit Nrq3

PA3019	-0,82	**-1,03**	-0,21	uup	probable ATP-binding component of ABC transporter
PA3023	1,27	**1,42**	0,15	yegS	conserved hypothetical protein
PA3032	**2,01**	**1,90**	-0,11	snr1	cytochrome c Snr1
PA3040	**1,50**	**1,62**	0,12	yqjD	conserved hypothetical protein
PA3042	0,99	**1,51**	0,52	NA	hypothetical protein
PA3049	**2,47**	**2,09**	-0,38	rmf	ribosome modulation factor
PA3089	1,00	**1,96**	0,96	NA	hypothetical protein
PA3162	**-1,56**	**-1,57**	-0,01	rpsA	30S ribosomal protein S1
PA3179	-0,72	**-1,14**	-0,42	yciL	conserved hypothetical protein
PA3181	**-3,77**	-0,99	**2,78**	edaA	2-keto-3-deoxy-6-phosphogluconate aldolase
PA3182	**-3,77**	-1,03	**2,74**	pgl	6-phosphogluconolactonase
PA3183	**-4,01**	**-1,09**	**2,92**	zwf	glucose-6-phosphate 1-dehydrogenase
PA3186	-0,15	**5,55**	**5,69**	oprB	Glucose/carbohydrate outer membrane porin OprB precursor
**PA3187**	**-2,96**	**2,74**	**5,70**	gltK	probable ATP-binding component of ABC transporter
**PA3188**	**-6,24**	-1,26	**4,98**	gltG	probable permease of ABC sugar transporter
**PA3189**	**-1,92**	-0,10	1,82	gltF	probable permease of ABC sugar transporter
PA3190	0,23	**5,83**	**5,60**	gltB	probable binding protein component of ABC sugar transporter
PA3191	**-1,32**	0,04	1,36	gtrS	glucose transport sensor, GtrS
PA3192	**-1,90**	-0,12	**1,78**	gltR	two-component response regulator GltR
PA3193	**-1,91**	-0,12	**1,79**	glk	glucokinase
PA3194	**-3,07**	-0,72	**2,35**	edd	phosphogluconate dehydratase
PA3195	**-3,04**	-0,58	**2,46**	gapA	glyceraldehyde 3-phosphate dehydrogenase
PA3229	**1,04**	**1,48**	0,45	NA	hypothetical protein
PA3236	**1,43**	1,11	-0,31	betX	BetX
PA3246	**-1,30**	**-1,27**	0,03	rluA	pseudouridine synthase RluA
PA3250	**1,78**	**1,47**	-0,31	NA	hypothetical protein
PA3251	0,92	**1,63**	0,71	NA	hypothetical protein
PA3263	**-1,14**	-0,78	0,37	yaiD	conserved hypothetical protein
PA3268	**-1,58**	-0,98	0,60	NA	probable TonB-dependent receptor
PA3274	1,63	**1,72**	0,08	NA	hypothetical protein
PA3308	-0,99	**-1,00**	-0,01	hepA	RNA helicase HepA
PA3309	**-1,20**	-0,41	0,79	uspK	conserved hypothetical protein
PA3311	**1,50**	**1,57**	0,07	nbdA	NbdA
PA3327	**1,34**	0,62	-0,72	NA	probable non-ribosomal peptide synthetase
PA3328	**1,61**	**1,13**	-0,47	NA	probable FAD-dependent monooxygenase
PA3329	**1,73**	**1,14**	-0,58	NA	hypothetical protein
PA3330	**1,67**	0,98	-0,70	NA	probable short chain dehydrogenase

PA3331	**1,80**	**1,20**	-0,60	NA	cytochrome P450
PA3332	**1,75**	**1,27**	-0,49	NA	conserved hypothetical protein
PA3333	**1,83**	**1,03**	-0,80	fabH2	3-oxoacyl-[acyl-carrier-protein] synthase III
PA3334	**2,19**	**1,32**	-0,87	NA	probable acyl carrier protein
PA3335	**1,70**	**1,13**	-0,57	NA	hypothetical protein
PA3336	**1,83**	1,04	-0,78	NA	probable major facilitator superfamily (MFS) transporter
PA3347	**1,15**	**1,17**	0,02	hsbA	HptB-dependent secretion and biofilm anti anti-sigma factor HsbA
PA3361	**2,37**	**1,56**	-0,81	lecB	fucose-binding lectin PA-IIL
PA3369	**1,57**	**1,49**	-0,08	NA	hypothetical protein
PA3415	**1,93**	1,53	-0,40	NA	probable dihydrolipoamide acetyltransferase
PA3416	**2,34**	**2,36**	0,02	NA	probable pyruvate dehydrogenase E1 component, beta chain
PA3417	**1,71**	**2,05**	0,33	NA	probable pyruvate dehydrogenase E1 component, alpha subunit
PA3418	**1,65**	**2,17**	0,52	ldh	leucine dehydrogenase
PA3432	**-1,67**	-0,73	0,94	NA	hypothetical protein
PA3451	**2,46**	**1,90**	-0,56	NA	hypothetical protein
PA3459	0,89	**1,23**	0,34	asnB	probable glutamine amidotransferase
PA3465	0,57	**1,35**	0,78	yfiS	conserved hypothetical protein
PA3477	**1,25**	0,89	-0,37	rhlR	transcriptional regulator RhlR
PA3478	**1,60**	**1,19**	-0,40	rhlB	rhamnosyltransferase chain B
PA3479	**1,74**	**1,31**	-0,43	rhlA	rhamnosyltransferase chain A
PA3484	-0,83	**-1,09**	-0,26	tse3	Tse3
PA3516	**1,21**	0,94	-0,27	NA	probable lyase
PA3519	**1,90**	**1,28**	-0,63	NA	hypothetical protein
PA3531	**-1,40**	**-1,36**	0,05	bfrB	bacterioferritin
PA3560	-0,39	**2,01**	**2,40**	fruA	phosphotransferase system transporter fructose-specific IIBC component, FruA
PA3561	0,16	**2,55**	**2,38**	fruK	1-phosphofructokinase
PA3562	-0,25	**2,53**	**2,78**	fruI	phosphotransferase system transporter enzyme I, FruI
PA3568	**1,93**	0,82	-1,11	ymmS	probable acetyl-coa synthetase
PA3569	**1,33**	0,42	-0,90	mmsB	3-hydroxyisobutyrate dehydrogenase
PA3570	**1,56**	0,87	-0,69	mmsA	methylmalonate-semialdehyde dehydrogenase
PA3621	**-1,27**	**-1,27**	0,00	fdxA	ferredoxin I
PA3622	**1,36**	**1,26**	-0,10	rpoS	sigma factor RpoS
PA3635	**-1,11**	**-1,13**	-0,02	eno	enolase
PA3640	-0,82	**-1,00**	-0,18	dnaE	DNA polymerase III, alpha chain
PA3641	**-1,78**	**-1,92**	-0,14	NA	probable amino acid permease
PA3645	-0,77	**-1,09**	-0,31	fabZ	(3R)-hydroxymyristoyl-[acyl carrier protein] dehydratase

PA3652	**-1,17**	**-1,23**	-0,06	uppS	undecaprenyl pyrophosphate synthetase
PA3653	**-1,05**	**-1,23**	-0,18	frr	ribosome recycling factor
PA3655	**-1,50**	**-1,41**	0,09	tsf	elongation factor Ts
PA3656	**-1,46**	**-1,52**	-0,06	rpsB	30S ribosomal protein S2
PA3675	**-1,09**	**-1,01**	0,08	NA	hypothetical protein
PA3688	1,06	**1,70**	0,63	NA	hypothetical protein
PA3691	**1,61**	**2,11**	0,50	NA	hypothetical protein
PA3692	**1,54**	**2,05**	0,50	lptF	Lipotoxon F, LptF
PA3700	**-1,40**	**-1,27**	0,13	lysS	lysyl-tRNA synthetase
PA3709	**1,46**	**1,51**	0,05	NA	probable major facilitator superfamily (MFS) transporter
PA3710	**1,36**	0,75	-0,62	NA	probable GMC-type oxidoreductase
PA3716	**-1,03**	**-1,04**	-0,01	NA	hypothetical protein
PA3723	**2,20**	**1,42**	-0,79	yqjM	probable FMN oxidoreductase
PA3724	**2,96**	**1,82**	-1,14	lasB	elastase LasB
PA3729	-0,99	**-1,21**	-0,22	NA	conserved hypothetical protein
PA3734	**1,65**	**1,64**	-0,01	NA	hypothetical protein
PA3742	**-1,53**	-1,05	0,48	rplS	50S ribosomal protein L19
PA3743	**-1,15**	**-1,35**	-0,20	trmD	tRNA (guanine-N1)-methyltransferase
PA3744	**-1,11**	**-1,32**	-0,22	rimM	16S rRNA processing protein
PA3745	**-1,35**	**-1,73**	-0,38	rpsP	30S ribosomal protein S16
PA3763	-0,99	**-1,04**	-0,05	purL	phosphoribosylformylglycinamidine synthase
PA3769	**-1,41**	**-1,27**	0,14	guaA	GMP synthase
PA3770	**-1,09**	**-1,17**	-0,08	guaB	inosine-5'-monophosphate dehydrogenase
PA3790	**-1,52**	**-1,68**	-0,16	oprC	Putative copper transport outer membrane porin OprC precursor
PA3795	0,93	**1,02**	0,08	NA	probable oxidoreductase
PA3807	**-1,02**	-0,76	0,26	ndk	nucleoside diphosphate kinase
PA3812	-0,78	**-1,17**	-0,39	iscA	probable iron-binding protein IscA
PA3818	**-1,06**	-0,89	0,17	suhB	extragenic suppressor protein SuhB
PA3821	**-1,58**	**-1,62**	-0,04	secD	secretion protein SecD
PA3822	-0,79	**-1,07**	-0,27	yajC	conserved hypothetical protein
PA3834	**-1,09**	-0,90	0,19	valS	valyl-tRNA synthetase
PA3858	**1,16**	0,86	-0,30	aapJ	probable amino acid-binding protein
PA3863	**-1,51**	-0,84	0,67	dauA	FAD-dependent catabolic D-arginine dehydrogenase, DauA
PA3890	0,95	**1,28**	0,33	opuCB	OpuC ABC transporter, permease protein, OpuCB
PA3891	0,91	**1,33**	0,42	opuCA	OpuC ABC transporter, ATP-binding protein, OpuCA
PA3892	-0,99	**-1,37**	-0,38	NA	conserved hypothetical protein
PA3919	**1,02**	**1,26**	0,23	ylaK	conserved hypothetical protein
PA3922	**2,37**	**1,95**	-0,42	NA	conserved hypothetical protein
PA3923	**2,11**	**1,53**	-0,58	NA	hypothetical protein

PA3924	**1,08**	0,90	-0,18	NA	probable medium-chain acyl-CoA ligase
PA3934	-0,55	**-1,51**	-0,96	NA	conserved hypothetical protein
PA3969a	-0,74	**-1,34**	-0,59	NA	NA
PA3980	-0,98	**-1,20**	-0,22	miaB; yleA	conserved hypothetical protein
PA3986	**1,50**	**1,58**	0,08	NA	hypothetical protein
PA4000	-0,63	**-1,07**	-0,44	rlpA	RlpA
PA4006	-0,87	**-1,07**	-0,20	nadD1,nadD	nicotinate mononucleotide adenylyltransferase NadD1
PA4015	**1,29**	**1,33**	0,04	NA	conserved hypothetical protein
PA4031	**-1,20**	**-1,16**	0,04	ppa	inorganic pyrophosphatase
PA4053	**-1,09**	**-1,34**	-0,25	ribE	6,7-dimethyl-8-ribityllumazine synthase
PA4061	**-1,03**	**-1,08**	-0,06	ybbN	probable thioredoxin
PA4067	**-1,43**	-0,75	0,69	oprG	Outer membrane protein OprG precursor
PA4078	**1,94**	**1,66**	-0,29	NA	probable nonribosomal peptide synthetase
PA4112	**1,56**	**1,37**	-0,19	NA	probable sensor/response regulator hybrid
PA4139	**2,23**	**1,83**	-0,40	NA	hypothetical protein
PA4140	**2,02**	**1,53**	-0,49	NA	hypothetical protein
PA4141	**2,00**	**1,69**	-0,31	NA	hypothetical protein
PA4142	**1,54**	1,17	-0,37	NA	probable secretion protein
PA4205	**1,69**	**1,90**	0,21	mexG	hypothetical protein
PA4206	**1,90**	**1,45**	-0,45	mexH	probable Resistance-Nodulation-Cell Division (RND) efflux membrane fusion protein precursor
PA4207	**1,59**	**1,24**	-0,34	mexI	probable Resistance-Nodulation-Cell Division (RND) efflux transporter
PA4209	**2,72**	**1,80**	-0,91	phzM	probable phenazine-specific methyltransferase
PA4210	**2,63**	**1,42**	-1,21	phzA1	probable phenazine biosynthesis protein
PA4211	**2,84**	**1,70**	-1,14	phzB1	probable phenazine biosynthesis protein
PA4212	**1,92**	0,96	-0,95	phzC1	phenazine biosynthesis protein PhzC
PA4217	**3,01**	**1,82**	-1,19	phzS	flavin-containing monooxygenase
PA4234	**-1,13**	-0,90	0,23	uvrA	excinuclease ABC subunit A
PA4237	**-1,08**	-0,57	0,51	rplQ	50S ribosomal protein L17
PA4238	**-1,43**	**-1,40**	0,03	rpoA	DNA-directed RNA polymerase alpha chain
PA4239	**-1,51**	**-1,62**	-0,11	rpsD	30S ribosomal protein S4
PA4240	**-1,20**	-0,92	0,28	rpsK	30S ribosomal protein S11
PA4241	**-1,40**	**-1,05**	0,36	rpsM	30S ribosomal protein S13
PA4242	**-1,32**	**-1,68**	-0,36	rpmJ	50S ribosomal protein L36
PA4243	**-1,62**	**-1,63**	0,00	secY	secretion protein SecY
PA4244	**-1,86**	**-1,96**	-0,10	rplO	50S ribosomal protein L15
PA4245	**-1,52**	**-1,59**	-0,08	rpmD	50S ribosomal protein L30
PA4246	**-1,68**	**-1,79**	-0,11	rpsE	30S ribosomal protein S5
PA4247	**-1,78**	**-1,68**	0,10	rplR	50S ribosomal protein L18

PA4248	**-1,84**	**-1,69**	0,16	rplF	50S ribosomal protein L6
PA4249	**-1,47**	**-1,36**	0,12	rpsH	30S ribosomal protein S8
PA4250	**-1,03**	-0,56	0,46	rpsN	30S ribosomal protein S14
PA4251	**-1,24**	**-1,09**	0,15	rplE	50S ribosomal protein L5
PA4252	**-1,46**	**-1,56**	-0,10	rplX	50S ribosomal protein L24
PA4253	**-1,12**	**-1,05**	0,07	rplN	50S ribosomal protein L14
PA4254	**-1,27**	**-1,10**	0,17	rpsQ	30S ribosomal protein S17
PA4255	**-1,47**	**-1,40**	0,07	rpmC	50S ribosomal protein L29
PA4256	**-1,36**	**-1,42**	-0,06	rplP	50S ribosomal protein L16
PA4257	**-1,49**	**-1,27**	0,22	rpsC	30S ribosomal protein S3
PA4258	**-1,65**	**-1,54**	0,11	rplV	50S ribosomal protein L22
PA4259	**-1,60**	**-1,75**	-0,14	rpsS	30S ribosomal protein S19
PA4260	**-1,78**	**-2,22**	-0,44	rplB	50S ribosomal protein L2
PA4261	**-2,01**	**-1,91**	0,10	rplW	50S ribosomal protein L23
PA4262	**-2,08**	**-2,16**	-0,08	rplD	50S ribosomal protein L4
PA4263	**-1,69**	**-1,55**	0,14	rplC	50S ribosomal protein L3
PA4264	**-1,77**	**-1,90**	-0,13	rpsJ	30S ribosomal protein S10
PA4265	**-1,45**	**-1,40**	0,04	tufA	elongation factor Tu
PA4266	**-1,47**	**-1,49**	-0,02	fusA1	elongation factor G
PA4267	**-1,35**	**-1,30**	0,06	rpsG	30S ribosomal protein S7
PA4268	**-1,56**	**-1,56**	0,00	rpsL	30S ribosomal protein S12
PA4269	**-1,27**	**-1,39**	-0,12	rpoC	DNA-directed RNA polymerase beta* chain
PA4270	**-1,12**	**-1,05**	0,07	rpoB	DNA-directed RNA polymerase beta chain
PA4271	**-1,64**	**-1,35**	0,30	rplL	50S ribosomal protein L7 / L12
PA4272	**-1,94**	**-2,03**	-0,09	rplJ	50S ribosomal protein L10
PA4273	**-1,72**	**-1,88**	-0,17	rplA	50S ribosomal protein L1
PA4274	**-1,62**	**-1,49**	0,13	rplK	50S ribosomal protein L11
PA4276	**-1,07**	**-1,36**	-0,29	secE	secretion protein SecE
PA4277	**-1,34**	**-1,35**	0,00	tufB	elongation factor Tu
PA4279	-0,96	**-1,09**	-0,13	NA	hypothetical protein
PA4280	-0,92	**-1,28**	-0,36	birA	BirA bifunctional protein
PA4292	**-1,25**	**-1,43**	-0,18	NA	probable phosphate transporter
PA4296	**1,88**	**1,31**	-0,57	pprB	two-component response regulator, PprB
PA4305	1,25	**1,56**	0,31	rcpC	RcpC
PA4311	**1,47**	**1,27**	-0,19	NA	conserved hypothetical protein
PA4333	**-1,12**	**-1,12**	0,00	fumA	probable fumarase
PA4362	**1,98**	**1,89**	-0,09	NA	hypothetical protein
PA4377	1,32	**1,56**	0,24	NA	hypothetical protein
PA4385	**-1,49**	**-1,39**	0,10	groEL	GroEL protein
PA4386	**-1,55**	**-1,51**	0,04	groES	GroES protein
PA4387	**-1,78**	**-2,11**	-0,33	fxsA	conserved hypothetical protein
PA4402	-0,98	**-1,36**	-0,39	argJ	glutamate N-acetyltransferase
PA4428	-1,13	**-1,19**	-0,06	sspA	stringent starvation protein A

PA4429	**-1,05**	**-1,02**	0,03	NA	probable cytochrome c1 precursor
PA4430	**-1,14**	**-1,15**	-0,01	NA	probable cytochrome b
PA4431	**-1,06**	**-1,01**	0,04	NA	probable iron-sulfur protein
PA4433	**-1,53**	**-1,53**	0,00	rplM	50S ribosomal protein L13
PA4438	**-1,03**	**-1,23**	-0,19	yhcM	conserved hypothetical protein
PA4443	**-1,15**	-0,67	0,48	cysD	ATP sulfurylase small subunit
PA4457	-0,73	**-1,04**	-0,31	kpsF; yrbH; kdsD	arabinose-5-phosphate isomerase KdsD
PA4458	-0,85	**-1,12**	-0,27	yrbI	conserved hypothetical protein
PA4463	0,69	**1,12**	0,43	yhbH	conserved hypothetical protein
PA4468	1,03	**1,76**	0,73	sodM	superoxide dismutase
PA4469	0,73	**1,53**	0,79	NA	hypothetical protein
PA4470	0,48	**1,61**	1,14	fumC1	fumarate hydratase
PA4471	**1,16**	**1,90**	0,73	fagA	hypothetical protein
PA4480	**-1,06**	**-1,13**	-0,06	mreC	rod shape-determining protein MreC
PA4481	-0,99	**-1,10**	-0,11	mreB	rod shape-determining protein MreB
PA4483	**-1,12**	**-1,14**	-0,01	gatA	Glu-tRNA(Gln) amidotransferase subunit A
PA4484	**-1,20**	**-1,20**	0,01	gatB	Glu-tRNA(Gln) amidotransferase subunit B
PA4496	**1,23**	-0,05	**-1,28**	dppA1	probable binding protein component of ABC transporter
PA4497	**1,43**	0,58	-0,84	dppA2	probable binding protein component of ABC transporter
PA4501	**1,48**	0,86	-0,62	opdD,opdP	Glycine-glutamate dipeptide porin OpdP
PA4542	**-1,68**	**-1,48**	0,20	clpB	ClpB protein
PA4566	**-1,02**	-1,00	0,03	obg	GTP-binding protein Obg
PA4568	**-1,53**	**-1,79**	-0,26	rplU	50S ribosomal protein L21
PA4572	**-1,20**	**-1,24**	-0,04	fklB	peptidyl-prolyl cis-trans isomerase FklB
PA4587	**-1,74**	**-1,59**	0,15	ccpR	cytochrome c551 peroxidase precursor
PA4588	0,36	**2,09**	**1,74**	gdhA	glutamate dehydrogenase
PA4590	**2,01**	**1,72**	-0,29	pra	protein activator
PA4602	**-1,10**	**-1,10**	0,00	glyA3	serine hydroxymethyltransferase
PA4607	**2,49**	**2,46**	-0,03	NA	hypothetical protein
PA4614	**1,23**	**1,85**	0,62	mscL	conductance mechanosensitive channel
PA4623	**1,20**	**1,28**	0,08	NA	hypothetical protein
PA4624	**1,04**	0,66	-0,37	cdrB	cyclic diguanylate-regulated TPS partner B, CdrB
PA4640	**-1,06**	**-1,28**	-0,22	mqoB	malate:quinone oxidoreductase
PA4645	-0,89	**-1,06**	-0,17	hpt; hprT	probable purine/pyrimidine phosphoribosyl transferase
PA4646	**-1,06**	**-1,21**	-0,15	upp	uracil phosphoribosyltransferase
PA4647	-0,81	**-1,06**	-0,25	uraA	uracil permease
PA4665	**-1,01**	**-1,03**	-0,02	prfA	peptide chain release factor 1
PA4670	**-1,13**	**-1,47**	-0,35	prs	ribose-phosphate pyrophosphokinase

PA4671	**-1,63**	**-1,60**	0,03	rplY	probable ribosomal protein L25
PA4672	-0,87	**-1,14**	-0,27	pth	peptidyl-tRNA hydrolase
PA4673	**-1,55**	**-1,21**	0,34	ychF	conserved hypothetical protein
PA4675	**-1,36**	-0,88	0,48	chtA	ChtA
PA4680	**1,69**	**1,78**	0,09	NA	hypothetical protein
PA4681	1,41	**1,52**	0,11	NA	hypothetical protein
PA4682	**1,39**	**1,23**	-0,16	NA	hypothetical protein
PA4684	-0,98	**-1,05**	-0,07	NA	hypothetical protein
PA4686	**-1,19**	-0,96	0,22	NA	hypothetical protein
PA4695	-0,85	**-1,03**	-0,18	ilvH	acetolactate synthase isozyme III small subunit
PA4709	0,06	**1,24**	1,18	phuS	PhuS
PA4710	-0,05	**1,26**	**1,31**	phuR	Heme/Hemoglobin uptake outer membrane receptor PhuR precursor
PA4720	**-1,56**	**-1,36**	0,20	trmA	tRNA (uracil-5-)-methyltransferase
PA4730	**-1,20**	**-1,09**	0,10	panC	pantoate--beta-alanine ligase
PA4738	**3,20**	**3,44**	0,24	yjbJ	conserved hypothetical protein
PA4739	**2,64**	**2,73**	0,09	NA	conserved hypothetical protein
PA4740	**-1,07**	**-1,02**	0,05	pnp	polyribonucleotide nucleotidyltransferase
PA4741	**-1,19**	-0,81	0,38	rpsO	30S ribosomal protein S15
PA4743	**-1,19**	-1,03	0,16	rbfA	ribosome-binding factor A
PA4744	**-1,51**	**-1,52**	-0,01	infB	translation initiation factor IF-2
PA4745	**-1,25**	**-1,33**	-0,08	nusA	N utilization substance protein A
PA4746	-0,69	**-1,21**	-0,52	yhbC	conserved hypothetical protein
PA4748	-0,54	**-1,00**	-0,47	tpiA	triosephosphate isomerase
PA4749	**-1,24**	**-1,24**	0,01	glmM	phosphoglucosamine mutase
PA4750	-1,01	**-1,23**	-0,21	folP	dihydropteroate synthase
PA4757	-0,87	**-1,00**	-0,13	yeaS	conserved hypothetical protein
PA4759	**-1,26**	**-1,21**	0,05	dapB	dihydrodipicolinate reductase
PA4760	**-1,57**	**-1,59**	-0,02	dnaJ	DnaJ protein
PA4761	**-2,02**	**-1,88**	0,14	dnaK	DnaK protein
PA4762	**-1,93**	**-1,86**	0,07	grpE	heat shock protein GrpE
PA4765	-0,91	**-1,20**	-0,29	omlA	Outer membrane lipoprotein OmlA precursor
PA4774	-0,94	**-1,08**	-0,13	NA	hypothetical protein
PA4781	**1,23**	1,03	-0,20	NA	cyclic di-GMP phosphodiesterase
PA4848	**-1,11**	-0,92	0,19	accC	biotin carboxylase
PA4852	**-1,13**	**-1,37**	-0,25	yhdG	conserved hypothetical protein
PA4854	**-1,39**	**-1,21**	0,18	purH	phosphoribosylaminoimidazolecarboxamide formyltransferase
PA4855	**-1,63**	**-1,23**	0,40	purD	phosphoribosylamine--glycine ligase
PA4876	**1,87**	**2,86**	0,99	osmE	osmotically inducible lipoprotein OsmE
PA4877	1,25	**1,71**	0,45	NA	hypothetical protein
PA4880	**1,20**	**1,23**	0,03	NA	probable bacterioferritin

PA4910	**1,52**	**1,54**	0,02	NA	branched chain amino acid ABC transporter ATP binding protein
PA4911	**1,61**	1,07	-0,54	NA	probable permease of ABC branched-chain amino acid transporter
PA4913	**1,96**	**1,40**	-0,56	NA	probable binding protein component of ABC transporter
PA4915	**1,56**	**1,17**	-0,40	NA	probable chemotaxis transducer
PA4918	**-1,92**	**-1,44**	0,48	pcnA	nicotinamidase, PcnA
PA4920	**-1,00**	-0,39	0,62	nadE	NH3-dependent NAD synthetase
PA4928	-0,79	**-1,04**	-0,25	ygiR; ygiQ	conserved hypothetical protein
PA4929	**1,56**	1,31	-0,25	NA	hypothetical protein
PA4932	**-1,34**	-0,92	0,42	rplI	50S ribosomal protein L9
PA4933	**-1,56**	**-1,44**	0,12	NA	hypothetical protein
PA4934	**-1,46**	**-1,07**	0,39	rpsR	30S ribosomal protein S18
PA4935	**-1,65**	**-1,77**	-0,12	rpsF	30S ribosomal protein S6
PA4943	**-1,08**	**-1,09**	-0,01	hflX	probable GTP-binding protein
PA4944	**-1,02**	-0,90	0,12	hfq	Hfq
PA5001	-0,98	**-1,02**	-0,04	ssg	cell surface-sugar biosynthetic glycosyltransferase, Ssg
PA5002	-0,77	**-1,06**	-0,29	dnpA	de-N-acetylase involved in persistence, DnpA
PA5009	-0,85	**-1,01**	-0,16	waaP	lipopolysaccharide kinase WaaP
PA5010	**-1,11**	**-1,19**	-0,08	waaG	UDP-glucose:(heptosyl) LPS alpha 1,3-glucosyltransferase WaaG
PA5015	**-1,46**	-0,98	0,47	aceE	pyruvate dehydrogenase
PA5016	**-1,55**	-0,99	0,56	aceF	dihydrolipoamide acetyltransferase
PA5036	0,11	**1,23**	1,12	gltB	glutamate synthase large chain precursor
PA5046	**-1,19**	**-1,19**	-0,01	NA	malic enzyme
PA5047	-0,89	**-1,03**	-0,15	NA	hypothetical protein
PA5049	**-1,44**	-0,86	0,58	rpmE	50S ribosomal protein L31
PA5052	**-1,41**	**-1,53**	-0,12	NA	hypothetical protein
PA5053	**-1,54**	**-1,64**	-0,10	hslV	heat shock protein HslV
PA5054	**-1,80**	**-1,54**	0,25	hslU	heat shock protein HslU
PA5058	**1,41**	**1,03**	-0,38	phaC2	poly(3-hydroxyalkanoic acid) synthase 2
PA5087	**-1,06**	**-1,44**	-0,38	NA	hypothetical protein
PA5088	**-1,57**	**-1,83**	-0,25	NA	hypothetical protein
PA5089	**-1,64**	**-2,11**	-0,47	pldB	PldB
PA5090	**-1,46**	**-1,92**	-0,46	vgrG5	VgrG5
PA5091	**-2,40**	**-2,65**	-0,25	hutG	N-formylglutamate amidohydrolase
PA5092	**-2,92**	**-3,18**	-0,27	hutI	imidazolone-5-propionate hydrolase HutI
PA5093	**-2,73**	**-3,06**	-0,33	NA	probable histidine/phenylalanine ammonia-lyase
PA5094	**-2,31**	**-2,60**	-0,29	NA	probable ATP-binding component of ABC transporter

PA5095	**-2,06**	**-2,44**	-0,38	NA	probable permease of ABC transporter
PA5096	**-1,81**	**-2,52**	-0,71	NA	probable binding protein component of ABC transporter
PA5097	**-2,06**	**-2,58**	-0,52	hutT	probable amino acid permease
PA5098	**-4,21**	**-4,74**	-0,54	hutH	histidine ammonia-lyase
PA5099	**-4,05**	**-4,73**	-0,68	NA	probable transporter
PA5100	**-5,11**	**-5,50**	-0,38	hutU	urocanase
PA5105	**-2,55**	**-2,79**	-0,24	hutC	histidine utilization repressor HutC
PA5106	**-3,54**	**-3,86**	-0,32	NA	conserved hypothetical protein
PA5117	**-1,34**	**-1,11**	0,23	typA	regulatory protein TypA
PA5118	**-1,31**	**-1,49**	-0,19	thiI	thiazole biosynthesis protein ThiI
PA5136	-0,88	**-1,34**	-0,45	NA	hypothetical protein
PA5139	**-1,38**	-1,12	0,26	NA	hypothetical protein
PA5152	-0,27	**-1,77**	**-1,50**	NA	probable ATP-binding component of ABC transporter
PA5153	0,26	-0,93	**-1,18**	NA	amino acid (lysine/arginine/ornithine/histidine/octopine) ABC transporter periplasmic binding protein
PA5154	-0,07	**-1,29**	-1,22	NA	probable permease of ABC transporter
PA5171	**-2,42**	**-2,38**	0,03	arcA	arginine deiminase
PA5172	**-2,59**	**-2,36**	0,23	arcB	ornithine carbamoyltransferase, catabolic
PA5173	**-2,74**	**-2,57**	0,18	arcC	carbamate kinase
PA5178	**1,23**	**1,51**	0,27	NA	conserved hypothetical protein
PA5180	**-1,66**	**-2,31**	-0,65	fdhD	conserved hypothetical protein
PA5181	**-1,63**	**-2,15**	-0,52	NA	probable oxidoreductase
PA5201	**-1,33**	**-1,36**	-0,02	yhgF; tex	conserved hypothetical protein
PA5203	**-1,02**	**-1,02**	0,00	gshA	glutamate--cysteine ligase
PA5212	1,05	**1,38**	0,33	NA	hypothetical protein
PA5219	**1,61**	0,89	-0,72	NA	hypothetical protein
PA5220	**1,96**	**1,27**	-0,69	NA	hypothetical protein
PA5239	**-1,01**	**-1,09**	-0,08	rho	transcription termination factor Rho
PA5261	0,84	**1,14**	0,30	algR	alginate biosynthesis regulatory protein AlgR
PA5298	**-1,17**	**-1,31**	-0,14	xpt	xanthine phosphoribosyltransferase
PA5303	**1,44**	1,17	-0,28	NA	conserved hypothetical protein
PA5304	0,81	**1,15**	0,34	dadA	D-amino acid dehydrogenase, small subunit
PA5316	**-1,38**	**-1,25**	0,14	rpmB	50S ribosomal protein L28
PA5340	**-1,10**	-0,91	0,19	NA	hypothetical protein
PA5348	**1,59**	**1,27**	-0,32	NA	probable DNA-binding protein
PA5359	**1,74**	**1,71**	-0,03	NA	hypothetical protein
PA5366	-0,65	**-1,19**	-0,54	pstB	ATP-binding component of ABC phosphate transporter
PA5380	**1,08**	0,62	-0,46	gbdR	GbdR
PA5396	**1,52**	0,88	-0,64	NA	hypothetical protein

PA5410	1,23	0,79	-0,44	gbcA	GbcA
PA5418	1,15	1,08	-0,07	soxA	sarcosine oxidase alpha subunit
PA5421	1,98	1,74	-0,24	fdhA	glutathione-independent formaldehyde dehydrogenase
PA5424	1,21	1,94	0,74	yeaQ	conserved hypothetical protein
PA5425	-1,20	-1,28	-0,08	purK	phosphoribosylaminoimidazole carboxylase
PA5426	-1,29	-1,48	-0,19	purE	phosphoribosylaminoimidazole carboxylase, catalytic subunit
PA5427	-1,36	-0,79	0,57	adhA	alcohol dehydrogenase
PA5435	-1,58	-0,81	0,77	oadA	probable transcarboxylase subunit
PA5436	-1,53	-0,56	0,97	NA	probable biotin carboxylase subunit of a transcarboxylase
PA5445	-1,40	-1,30	0,10	psecoA	probable coenzyme A transferase
PA5479	-1,48	-1,76	-0,28	gltP	proton-glutamate symporter
PA5481	2,43	2,40	-0,03	NA	hypothetical protein
PA5482	2,20	1,78	-0,42	NA	hypothetical protein
PA5490	-1,09	-1,31	-0,22	cc4	cytochrome c4 precursor
PA5502	-0,53	-1,26	-0,73	NA	hypothetical protein
PA5504	-1,04	-1,05	-0,01	NA	D-methionine ABC transporter membrane protein
PA5544	1,26	0,93	-0,33	NA	conserved hypothetical protein
PA5545	1,39	1,11	-0,28	NA	conserved hypothetical protein
PA5546	1,24	0,96	-0,29	NA	conserved hypothetical protein
PA5556	-1,01	-1,08	-0,07	atpA	ATP synthase alpha chain
PA5557	-1,00	-1,22	-0,23	atpH	ATP synthase delta chain
PA5559	-0,93	-1,07	-0,13	atpE	atp synthase C chain
PA5560	-0,81	-1,05	-0,24	atpB	ATP synthase A chain
PA5562	-0,98	-1,15	-0,16	spoOJ	chromosome partitioning protein Spo0J
PA5564	-1,36	-1,30	0,06	gidB	glucose inhibited division protein B
PA5568	-1,74	-1,77	-0,03	yidC	conserved hypothetical protein

[1]Red figures, significant difference (Padj<0.05)
[2]Boldface characters, deleted genes

	General sequencing statistics					
	Raw reads	Mapped reads	HQ reads	HQ%	Strand Specific reads on known transcripts	SS%
PAO1+GLU A	2858342	2774119	2665486	93,25%	2405388	84,15%
PAO1+GLU B	3612457	3499804	3263219	90,33%	2957221	81,86%
PAO1 A	3849630	3751918	3585149	93,13%	3167795	82,29%
PAO1 B	2965373	2917262	2793833	94,22%	2530642	85,34%
GUN+GLU A	3244245	3141297	2920182	90,01%	2528829	77,95%
GUN+GLU B	3116320	2976641	2795184	89,70%	2469372	79,24%

	rRNA removal evaluation			Strand specificity evaluation		
	mRNA%	rRNA%	tRNA %	mRNA SS%	rRNA SS%	tRNA SS%
PAO1+GLU A	68,26	0,23	0,54	94,94	83,77	98,47
PAO1+GLU B	72,16	0,56	0,77	93,94	78,33	98,47
PAO1 A	68,50	0,74	0,83	93,38	89,61	98,26
PAO1 B	75,71	0,24	0,44	95,12	88,25	98,18
GUN+GLU A	70,90	0,26	0,74	90,01	69,99	96,85
GUN+GLU B	58,64	2,30	0,68	91,04	97,13	96,30

*evaluation performed on mapped reads

	CDS strand specific coverage statitsics		
	Not expressed %	Reads covering 90% of the transcripts	Transcripts covered for half of their length (%)
PAO1+GLU A	2,6	4	76,2
PAO1+GLU B	1,3	8	86,0
PAO1 A	1,5	7	84,1
PAO1 B	2,1	4	77,3
GUN+GLU A	1,1	13	91,9
GUN+GLU B	1,4	8	84,1

Supplementary Table S3. Glucose responsive genes in PAO1

Locus	Log2 FC[a]			Name	Description	Regulator[b]
	PAO1+ *vs.* PAO1	GUN+ *vs.* PAO1+	GUN+ vs. PAO1			
**PA0888**	-1.31	-1.49	-2.80	*aotJ*	arginine/ornithine ABC transporter	ArgR[25]
**PA2264**	1.75	-1.13	0.62		hypothetical protein	PtxS[26]
**PA2265**	1.77	-0.84	0.93	*gad*	gluconate dehydrogenase	PtxS[26]
**PA2290**	1.68	-0.48	1.20	*gcd*	glucose dehydrogenase	nd
**PA2291**	1.88	-0.09	1.79		glucose-sensitive porin	nd
**PA2320**	1.88	-1.67	0.20	*gntR*	GntR transcriptional regulator	GntR[26]
**PA2321**	5.28	-5.62	-0.34	*gntK*	gluconokinase	nd
**PA2322**	5.15	-5.87	-0.72	*gntP*	gluconate permease	GntR[26]
**PA2323**	4.71	-1.62	3.09	*gapN*	glyceraldehyde-3-phosphate dehydrogenase	nd
**PA3181**	2.78	-3.77	-0.99	*edaA*	2-dehydro-3-deoxy-phosphogluconate aldolase	HexR[26]
**PA3182**	2.74	-3.77	-1.03	*pgl*	6-phosphogluconolactonase	HexR[26]
**PA3183**	2.92	-4.01	-1.09	*zwf*	glucose-6-phosphate 1-dehydrogenase	HexR[26]
**PA3186**	5.69	-0.15	5.55	*oprB*	porin B	GltR[26]
**PA3187**	5.70	-2.96	2.74	*gltK*	ABC transporter ATP-binding protein	GltR[26]
**PA3188**	4.98	-6.24	-1.26	*gltG*	sugar ABC transporter permease	GltR[26]
**PA3190**	5.60	0.23	5.83	*gltB*	sugar ABC transporter substrate-binding protein	GltR[26]
**PA3192**	1.78	-1.90	-0.12	*gltR*	response regulator GltR	nd
**PA3193**	1.79	-1.91	-0.12	*glk*	glucokinase	nd
**PA3194**	2.35	-3.07	-0.72	*edd*	phosphogluconate dehydratase	nd
**PA3195**	2.46	-3.04	-0.58	*gapA*	glyceraldehyde 3-phosphate dehydrogenase	nd
**PA3560**	2.40	-0.39	2.01	*fruA*	PTS system fructose-specific transporter subunit IIBC	FruR[27]
**PA3561**	2.38	0.16	2.55	*fruK*	1-phosphofructokinase	FruR[27]
**PA3562**	2.78	-0.25	2.53	*fruI*	PTS system fructose-specific transporter subunit FruI	FruR[27]
**PA4496**	-1.28	1.23	-0.05	*dppA1*	ABC transporter	nd
**PA4588**	1.74	0.36	2.09	*gdhA*	glutamate dehydrogenase	ArgR[25]
**PA4710**	1.31	-0.05	1.26	*phuR*	heme/hemoglobin uptake outer membrane receptor PhuR	Fur[28]
**PA5152**	-1.50	-0.27	-1.77		ABC transporter ATP-binding protein	ArgR[25]
**PA5153**	-1.18	0.26	-0.93		amino acid ABC transporter	ArgR[25]

[a]Red figures indicate statistically significant difference (Padj<0.05)
[b]Superscripts refer to bibliographic references. nd, not determined

Supplementary Table S4. DEGs encoding putative/known transcription regulators

Locus	Log2 FC[1]			Name	Description[2]
	PAO1+ *vs.* PAO1	GUN+ *vs.* PAO1+	GUN+ vs. PAO1		
PA0179	-0.34	1.44	1.10		TC response regulator
PA0527	0.82	-1.46	-0.64	*dnr*	transcriptional regulator Dnr
PA0547	-0.19	1.23	1.04		transcriptional regulator
PA0576	0.09	-1.21	-1.12	*rpoD*	sigma factor RpoD
PA0708	-0.25	1.90	1.65		transcriptional regulator
PA0893	-0.69	-0.69	-1.39	*argR*	transcriptional regulator ArgR
PA1196	0.86	-1.46	-0.61	*ddaR*	transcriptional regulator DdaR
PA2177	0.35	1.07	1.42		sensor/response regulator hybrid protein
PA2259[3]	1.55	-1.07	0.48	*ptxS*	PtxS transcriptional regulator
PA2320	1.88	-1.67	0.20	*gntR*	GntR transcriptional regulator
PA2572	-0.05	1.51	1.46		TC response regulator
PA3192	1.78	-1.90	-0.12	*gltR*	TC response regulator GltR
PA3308	-0.01	-0.99	-1.00	*hepA*	RNA polymerase-associated protein RapA
PA3477	-0.37	1.25	0.89	*rhlR*	transcriptional regulator RhlR
PA3622	-0.10	1.36	1.26	*rpoS*	sigma factor RpoS
PA4112	-0.19	1.56	1.37		sensor/response regulator hybrid protein
PA4296	-0.57	1.88	1.31	*pprB*	TC response regulator PprB
PA5105	-0.24	-2.55	-2.79	*hutC*	histidine utilization repressor HutC
PA5261	0.30	0.84	1.14	*algR*	TC response regulator AlgR
PA5380	-0.46	1.08	0.62	*gbdR*	protein GbdR

[1]Red figures indicate statistically significant difference (Padj<0.05)
[2]TC, Two-Component
[3]differential expression validated by qPCR

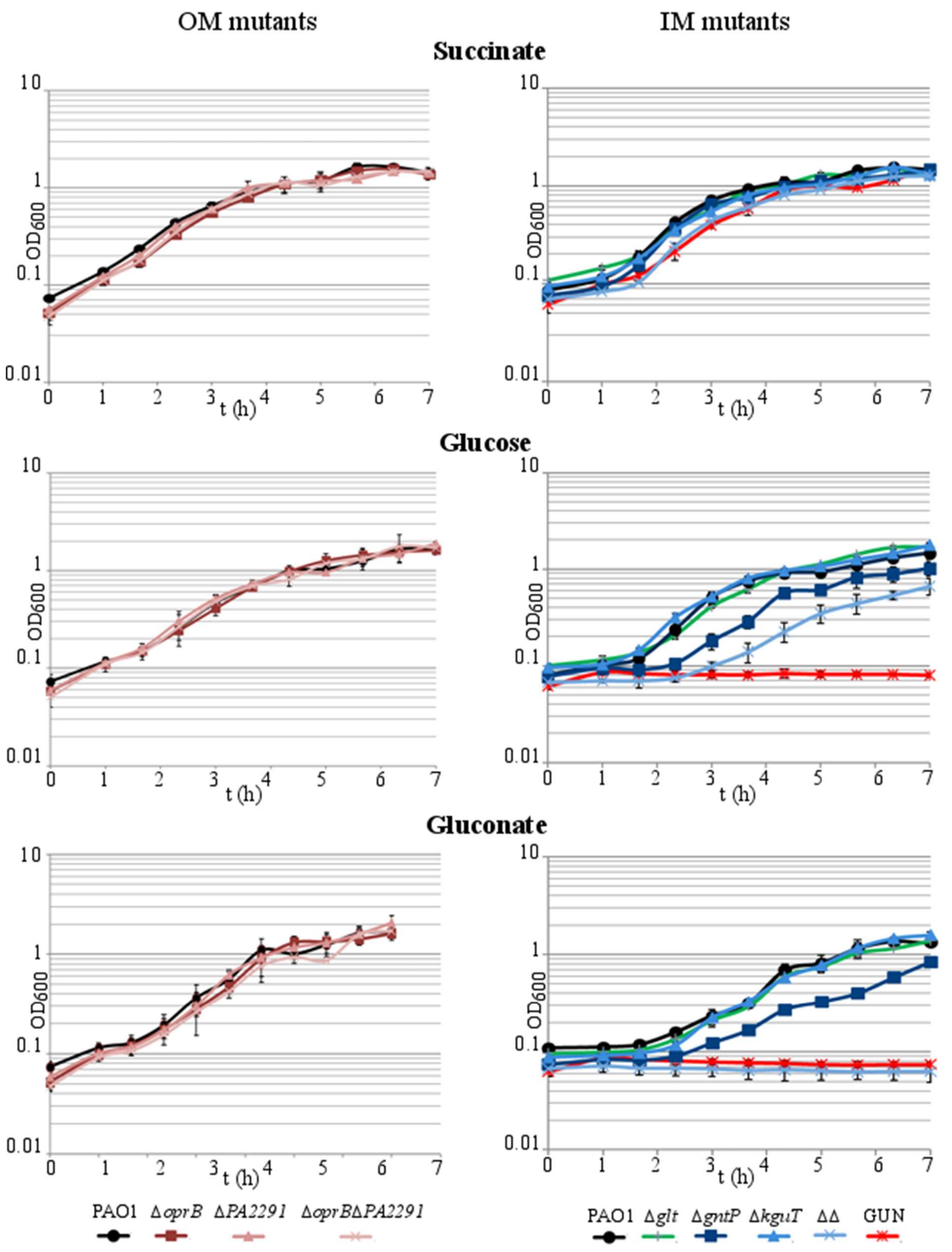

Fig. S1. Growth curves of glucose uptake mutants with different carbon source. Cultures of PAO1 and the indicated mutant strains were inoculated at the same OD_{600} in M9-Triton X-100 supplemented with 0.4% glucose, 0.4% gluconate or 0.5% succinate. The growth at 37°C was monitored every 40 min for 7 h. Symbols represent average (n=2) with SD of bacterial cell density (OD_{600}) at different time points. Generation time of PAO1 and IM mutants growing in the above media is reported in Table 1.

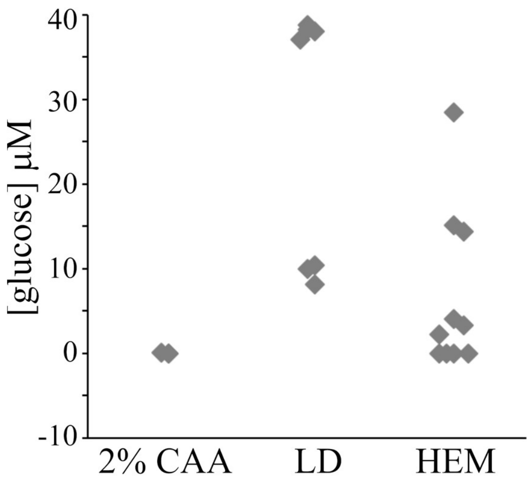

Fig. S2. Glucose concentration in bacterial media and *G. mellonella* hemolymph. Glucose concentration was estimated on 2% casamino acid stock solution (CAA; n=3); LD broth (LD; n=7) and *G. mellonella* hemolymph (HEM; n=10, each sample being a mix composed by 30 µl aliquots of hemolymph of 5 larvae). Diamonds indicate glucose concentration in individual samples.

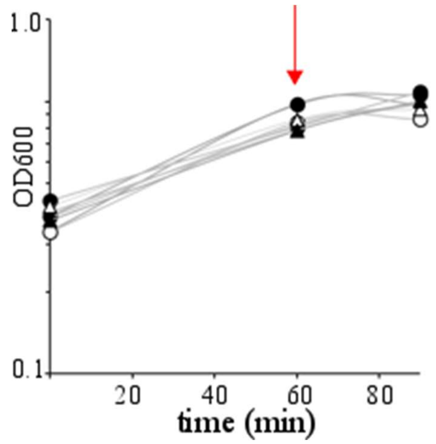

Fig. S3. Growth of cultures for RNA-Seq analysis. Independent cultures of PAO1 and GUN were grown in M9-CAA up to OD_{600}= 0.4. Glucose (0.4% (w/v) final concentration) was added and incubation at 37°C was protracted for 60 min before sampling the cultures for RNA extraction (red arrow). Triangle, PAO1; circle, GUN mutant. Empty symbols, no glucose added; black symbols, cultures supplemented with glucose.

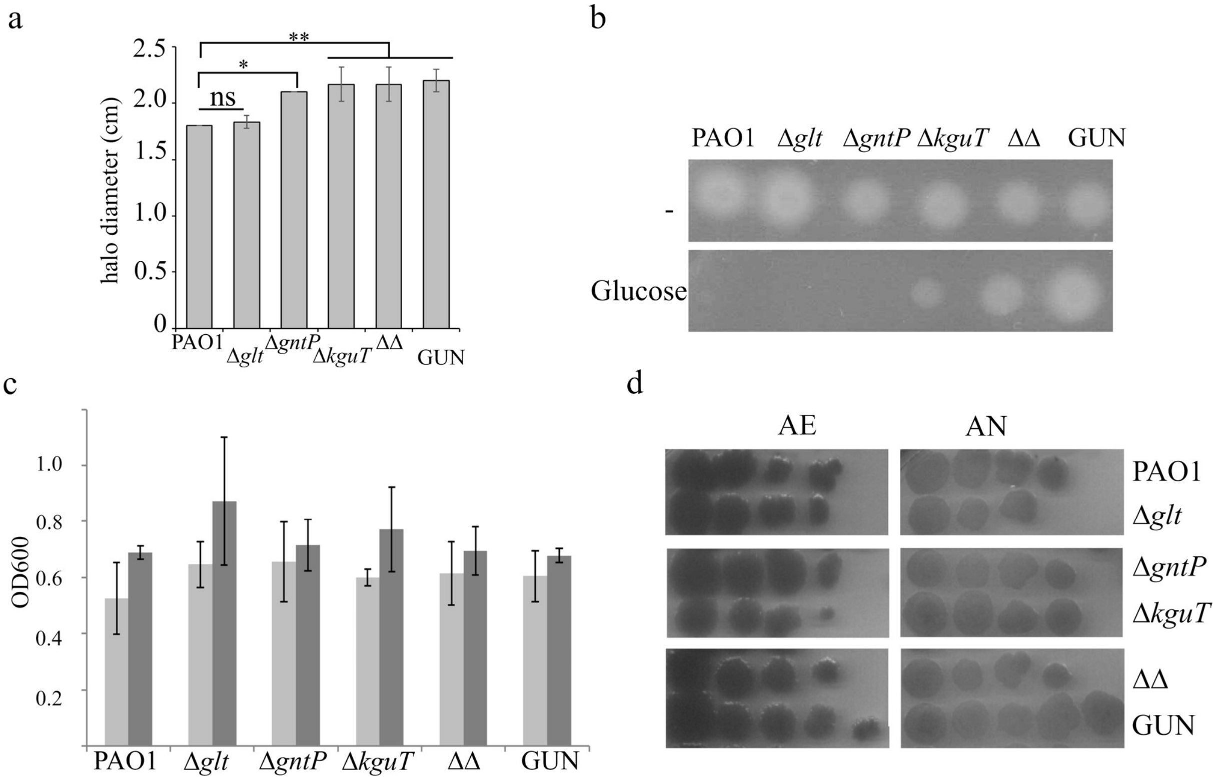

Fig. S4. *In vitro* phenotypic assays on glucose uptake mutants. In all panels, ΔΔ indicates PAO1 Δ*gntP* Δ*kguT* strain. Rhamnolipids (a) were measured as described in Supplementary Experimental procedures. Bars represent average (n=3) with SD. Significance was estimated with one-way Anova and Tukey post-hoc analysis, only the results of comparisons with PAO1 are reported (*, P<0.05; **, P<0.01; ns, not significant). Differences among Δ*kguT*, ΔΔ and GUN strains were not significant. b. Extracellular proteases from supernatants of cultures grown 17 h at 37°C in M9-CAA not supplemented (-) or supplemented with 0.4% glucose were detected by casein diffusion plate assay. The secretion of proteases is indicated by the formation of a white precipitate resulting from the hydrolysis of soluble casein to the insoluble *para*-casein derivative. The cultures were diluted to the same OD_{600} before the test. Three replicates gave comparable results. c. Growth in microaerophilic conditions. Light grey bars, cultures grown in M9-CAA; dark grey bars, cultures grown in LD. Bars represent average (n=3) with standard deviation. According to one-way Anova analysis, growth differences were not statistically significant at 0.05 level. d. Growth in anaerobiosis. Cultures were plated on LD-agar plates supplemented with 100 mM KNO_3 and incubated at 37 °C in aerobiosis (AE) for 16h or in anaerobiosis (AN) for 48h.

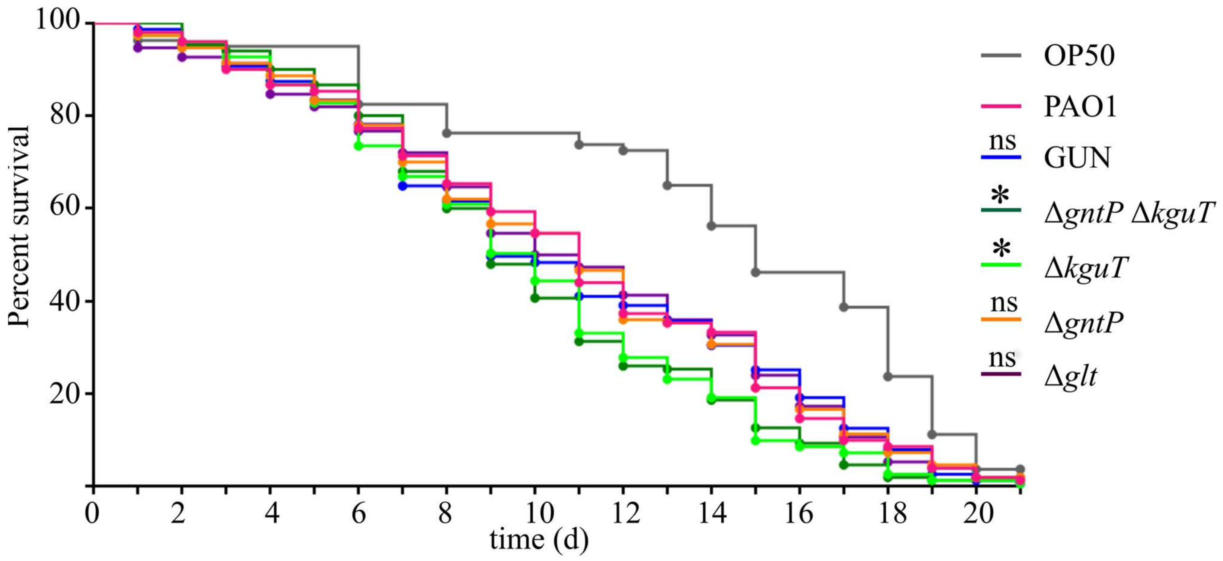

Fig. S5. *C. elegans* infection assay. Survival curves of *C. elegans* infected with glucose uptake mutants. Kaplan-Meyer curves represent results deriving from 3 independent experiments in which groups of 50 worms were fed on the indicated strains. OP50, *E. coli* strain. d, days post-infection. Significance was estimated with log-rank test; only the results relative to PAO1 are reported (*, P< 0.05; ns, not significant).

a

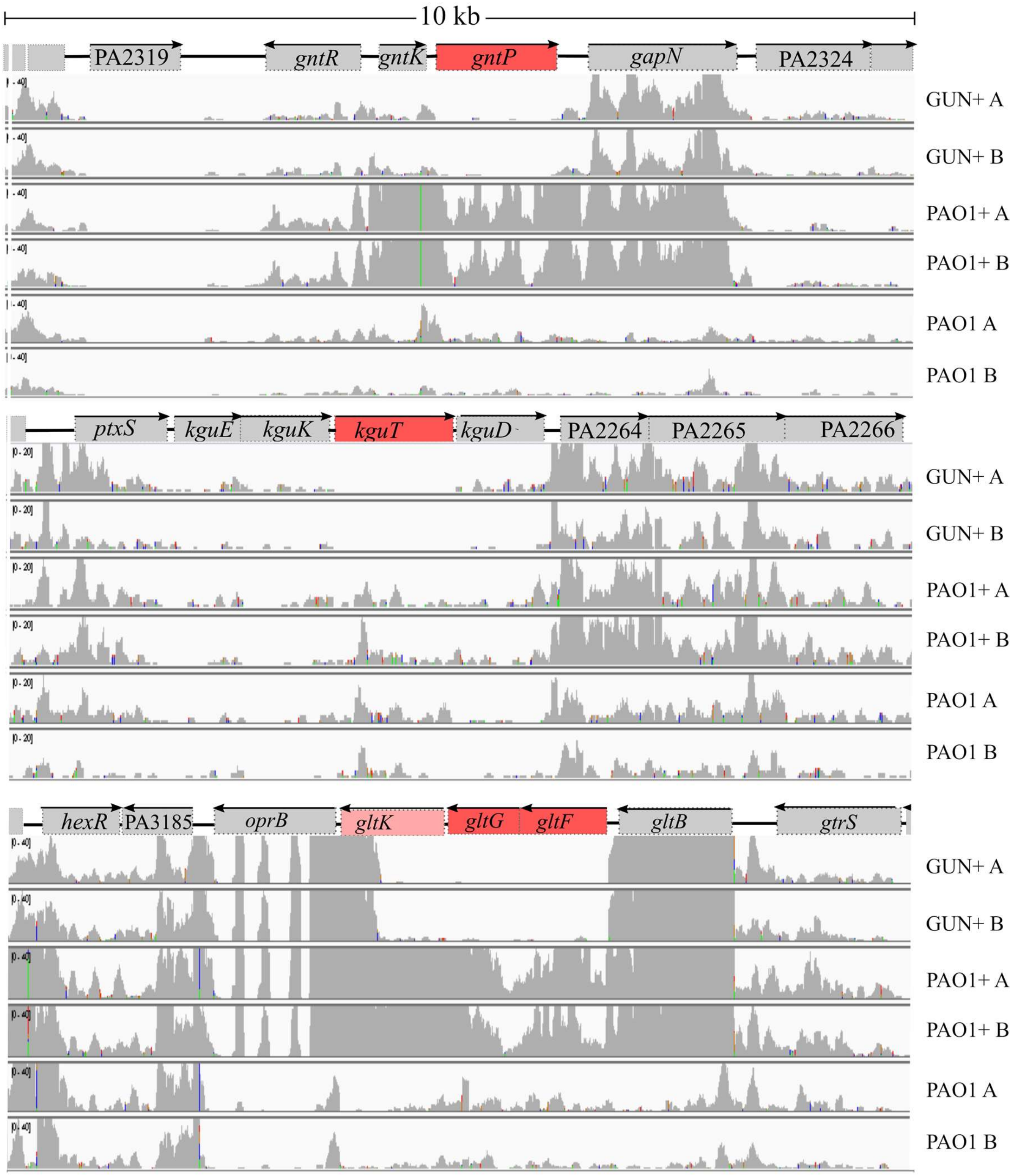

10 kb
PA2319
gntR
gntK
gntP
gapN
PA2324
GUN+ A
GUN+ B
PAO1+ A
PAO1+ B
PAO1 A
PAO1 B
ptxS
kguE
kguK
kguT
kguD
PA2264
PA2265
PA2266
GUN+ A
GUN+ B
PAO1+ A
PAO1+ B
PAO1 A
PAO1 B
hexR
PA3185
oprB
gltK
gltG
gltF
gltB
gtrS
GUN+ A
GUN+ B
PAO1+ A
PAO1+ B
PAO1 A
PAO1 B

b

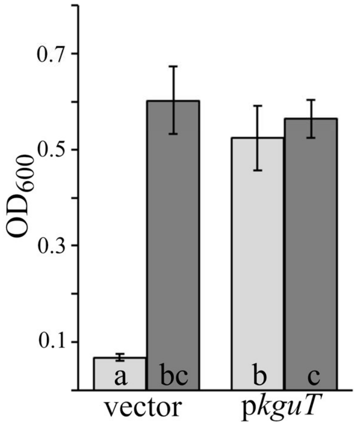

OD600
0.7
0.5
0.3
0.1
a
bc
b
c
vector
pkguT

Fig. S6. Polarity analysis of glucose uptake genes deletions. a. Transcriptional landscape of genomic regions encompassing the loci deleted in glucose uptake mutants. Illumina whole transcriptome reads per base in different strains/ conditions are reported below the map of the 10 kbp long genomic regions corresponding to deleted loci. Boxes, ORFs; lines, intergenic regions; arrows on top of boxes indicate the transcription direction. +, cultures supplemented with glucose; A and B, replicate samples analysed in the RNA-Seq experiment. b. Δ*kguT* mutation complementation assay. Cultures of PAO1 Δ*kguT* carrying either pGM931 (vector) or pGM2071 (p*kguT*) were inoculated in M9-Triton X-100-carbenicillin-arabinose supplemented with 0.1% 2-ketogluconate (light grey bars) or 0.5% succinate (dark grey bars). The cultures were incubated at 37°C and the OD_{600} was measured after 24 h. The bars represent average (n=3) with SD. Significance was evaluated with one-way Anova and Tukey post hoc test. Differences between means that share a letter (on the columns) are not statistically significant at 0.05 level.
